# Alkali metal cations modulate the geometry of different binding sites in HCN4 selectivity filter for permeation or block

**DOI:** 10.1085/jgp.202313364

**Published:** 2023-07-31

**Authors:** Jan H. Krumbach, Daniel Bauer, Atiyeh Sadat Sharifzadeh, Andrea Saponaro, Rene Lautenschläger, Kristina Lange, Oliver Rauh, Dario DiFrancesco, Anna Moroni, Gerhard Thiel, Kay Hamacher

**Affiliations:** 1 Department of Biology, Technical University of Darmstadt, Darmstadt, Germany; 2 Department of Biosciences, University of Milan, Milan, Italy; 3 Department of Physics, Technical University of Darmstadt, Darmstadt, Germany

## Abstract

Hyperpolarization-activated cyclic-nucleotide gated (HCN) channels are important for timing biological processes like heartbeat and neuronal firing. Their weak cation selectivity is determined by a filter domain with only two binding sites for K^+^ and one for Na^+^. The latter acts as a weak blocker, which is released in combination with a dynamic widening of the filter by K^+^ ions, giving rise to a mixed K^+^/Na^+^ current. Here, we apply molecular dynamics simulations to systematically investigate the interactions of five alkali metal cations with the filter of the open HCN4 pore. Simulations recapitulate experimental data like a low Li^+^ permeability, considerable Rb^+^ conductance, a block by Cs^+^ as well as a punch through of Cs^+^ ions at high negative voltages. Differential binding of the cation species in specific filter sites is associated with structural adaptations of filter residues. This gives rise to ion coordination by a cation-characteristic number of oxygen atoms from the filter backbone and solvent. This ion/protein interplay prevents Li^+^, but not Na^+^, from entry into and further passage through the filter. The site equivalent to S3 in K^+^ channels emerges as a preferential binding and presumably blocking site for Cs^+^. Collectively, the data suggest that the weak cation selectivity of HCN channels and their block by Cs^+^ are determined by restrained cation-generated rearrangements of flexible filter residues.

## Teaser

Krumbach et al. use molecular dynamics simulations to study the open HCN4 pore with different monovalent cations. Their simulations recapitulate experimental findings on cation-specific permeation and blocking of the channel and stress a mutual adaptation of cations and filter geometry as the basis for cation selectivity.

## Introduction

Hyperpolarization-activated cyclic nucleotide-gated (HCNs) channels generate the so-called funny currents (I_f_, I_h_) at negative voltages, which in turn regulate the free-running membrane potential as well as autonomous and rhythmic activity in cardiac myocytes (I_f_) and neurons (I_h_). It is long known that the primary amino acid sequence of these channels is very similar to one of the respective domains of highly selective K^+^ channels at the level of the selectivity filter (SF). Extensive electrophysiological characterization of HCN channels has shown that they also share some features with selective K^+^ channels including a neglectable conductance of Li^+^ and an appreciable conductance of Rb^+^ (DiFrancesco, 1982). But despite the similarities in the SF sequence, HCN channels exhibit distinct differences in their cation selectivity from canonical K^+^ channels. Most importantly, while canonical potassium channels transport K^+^ with a high preference over Na^+^, HCN channels exhibit only a weak selectivity for K^+^ over Na^+^ (Robinson and Siegelbaum, 2003). This peculiar feature is important for the physiological function of HCN channels as it guarantees an influx of Na^+^ ions sufficient to depolarize cells at negative voltages. Furthermore, while most K^+^ channels are blocked by Ba^2+^ and Cs^+^, HCN channels exhibit only a weak sensitivity to Ba^2+^ but a high sensitivity to Cs^+^ (Biel et al., 2009; DiFrancesco, 1982).

**Figure 1.**
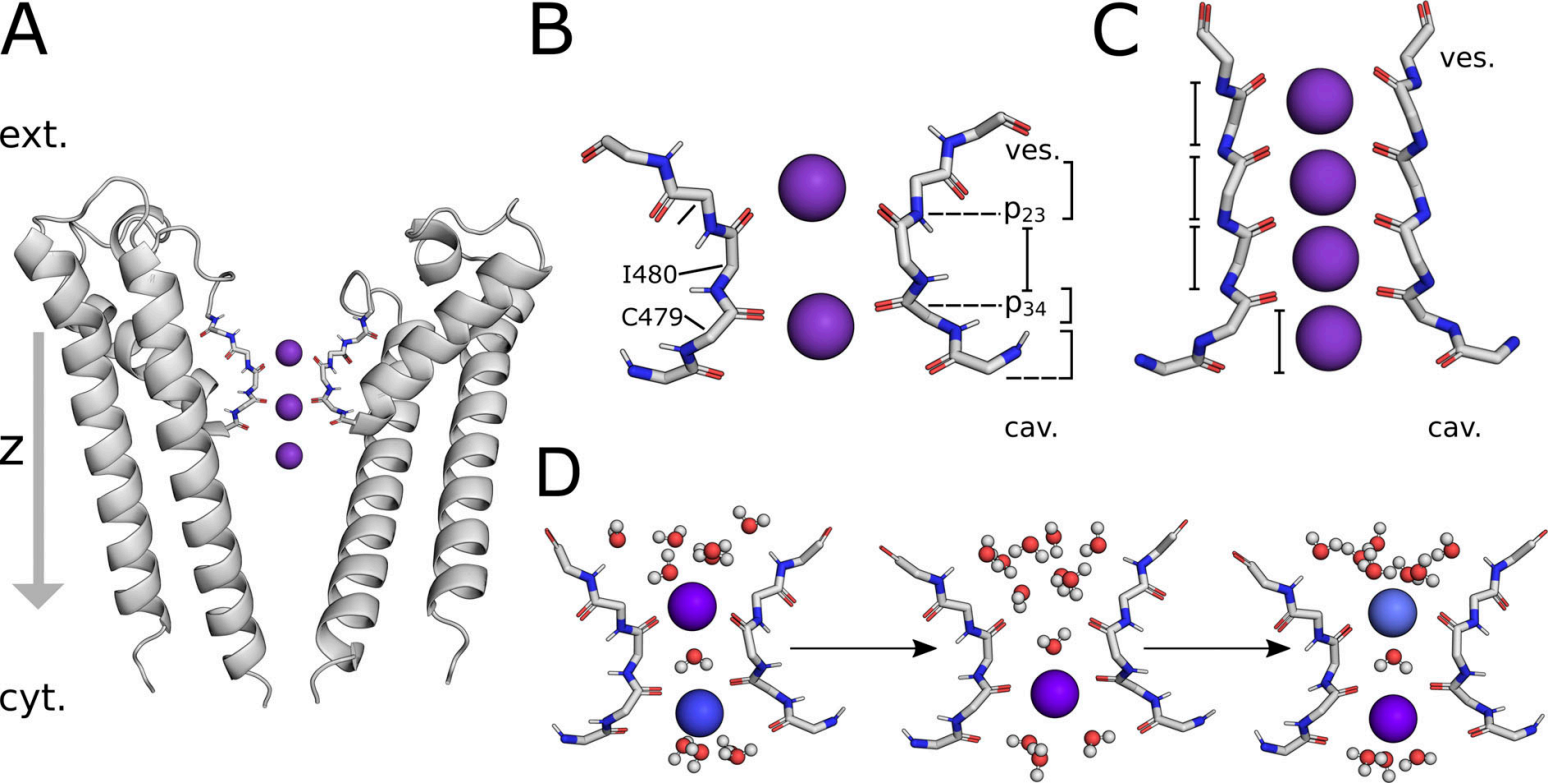
**Architecture of HCN4 channel pore and filter binding sites for K^+^ ions. (A)** Cross-section of the HCN4 PD in cartoon representation (PDB ID 7NP3). For clarity, only two opposing subunits of the homotetramer and ions K^+^ bound to the SF are shown. The main channel axis, centered in the pore and perpendicular to the membrane surface, is outlined as a z-vector pointing from the extracellular side (ext.) toward the cytosol (cyt.). **(B)** Schematic representation of the HCN4 SF. Carbonyl oxygen planes constituting the SF are marked as p23 (I480-CO) and p34 (I479-CO), and are indicated as black dashed lines. The three main binding sites for K^+^ are marked as Sa, Sb, and Sc; S3 refers to the respective K^+^ binding site in canonical K^+^ channels (Zhou et al., 2001). **(C)** Data here shows comparison of the selectivity filter of the canonical channel KcsA (PDB ID 1K4C). The pore region above and below the SF are termed as vestibule (ves.) and cavity (cav), respectively. **(D)** Detailed depiction of K^+^ conductance described in previous MD studies (Saponaro et al., 2021a). The protein backbone of SF residues is depicted in stick representation in all subplots, with hydrogen, carbon, nitrogen, and oxygen atoms colored in light gray, gray, blue, and red, respectively.

Recent high-resolution cryo-electron microscopy (cryoEM) structures of isoforms HCN1 and HCN4 have uncovered major differences between these channels and canonical K^+^ channels in the architecture of their selectivity filters (Lee and MacKinnon, 2017; Saponaro et al., 2021a). While the selectivity filter of K^+^-selective channels provides four distinct binding sites for K^+^ (S1 to S4), only one effective binding site in S3 is left in the filter of HCN channels (Fig. 1, A–C). This is because the tyrosine (Y482 in HCN4) side chain of the conserved GYG sequence is in the HCN4 pore 180°C rotated compared with that of canonical K^+^ channels. Also, the carbonyl oxygens of the filter Gly (G483), which form S2 in a canonical K^+^ channel, are rotated out of the central pore axis generating a wide vestibule (ves.) at the filter entrance (Fig. 1, B and C). Additionally, a conserved Thr in the filter sequence of selective K^+^ channels (TVGYG) is in HCN channels replaced by cysteines (CIGYG, C479 in HCN4). Consequently, without the contribution of the OH group of the threonine side chain, the typical S4 binding site of canonical K^+^ channels widens in HCN channels since passing ions are restricted to interact with the more distant carbonyl oxygen group. As a result, half of the binding site is contributed to by water molecules, which reduces the tight interaction of the protein with permeating cations.

With molecular dynamics (MD) simulations on the structure of the open HNC4 pore domain (PD), we were able to recapitulate and explain in molecular terms the major functional features of HCN channels known from experiments (Bauer et al., 2022; Saponaro et al., 2021a). This includes the low unitary conductance (1 pS) of HCN channels as well as their low selectivity ratio between K^+^ and Na^+^. Further scrutiny of the simulations uncovered many unique features of the HCN4 filter domain, which are different from both selective K^+^ channels as well as from the non-selective NaK channel (Bauer et al., 2022).

Previous simulations identified in the HCN4 selectivity filter the three main binding sites for K^+^ ions shown in Fig. 1 D, denoted here as Sa, Sb, and Sc (Saponaro et al., 2021a): Sa encompasses the carbonyl oxygen plane p23 constituted by carbonyl oxygens from I480 and the lower vestibule toward the extracellular filter entrance; Sb is located at the height of oxygen plane p34 formed by carbonyl oxygens from C479, whereas Sc is directly below p34 within the widened S4 site (Fig. 1 B). A K^+^ ion permeates when it moves from site Sa into Sb. This promotes the transition from c into the cavity, completing the permeation step and leaving the filter in a {Sb} “one ion” condition. Entry of a second ion into site a kicks the ion from position Sb into Sc, resetting the initial {Sa, Sc} ‘‘two ion’’ configuration. Coordinated by both carbonyl oxygens and proximate H_2_O molecules, K^+^ cations are positioned slightly above and below the oxygen planes in {Sa, Sc}. In {Sb}, the K^+^ ion is occupying the plane of carbonyl oxygens of p34, while water molecules from above and below stabilize this conformation by partial solvation. A site equivalent to S3 in canonical K^+^ channels, in which the K^+^ ion is coordinated by a cage of carbonyls from the eight filter amino acids (Zhou et al., 2001), is only briefly visited by K^+^ in the transition from position Sa to Sb. In mixed K^+^/Na^+^ solutions, which promote the conductance of both cations, Na^+^ ions bind to the same positions Sa and Sb as K^+^ ions.

Experimental studies have shown that the native I_f_/I_h_ currents and the cloned HCN channels exhibit distinct conduction features not only for physiologically relevant K^+^ and Na^+^ ions but also for the three remaining monovalent metal cations Li^+^, Rb^+^, and Cs^+^. The smallest Li^+^ ion is, with respect to K^+^, not (Ho et al., 1994) or only weakly transported. Therefore, reported permeability values for Li (P_Li_) relative to K^+^ (P_K_) are in the order of P_Li_/P_K_ = 0.02 and 0.03 for HCN1 or HCN4, respectively (Azene et al., 2003; D’Avanzo et al., 2009). It further occurs that the native I_f_ currents are not only impermeant to Li^+^ but also not blocked by this small cation (Ho et al., 1994). Rb^+^ on the other hand is well transported with P_Rb_/P_K_ ratios of 0.35 in the native I_f_ (Ho et al., 1994) and 0.48 in HCN4 (D’Avanzo et al., 2009). But despite a similar permeability, the mechanism of Rb^+^ permeation must be different from K^+^ because the former inhibits at negative voltages the conduction of K^+^ ions in a substantially voltage-independent manner (DiFrancesco, 1982). Also, Cs^+^ is interesting for understanding the permeation mechanism in HCN channels. It has been reported that this ion is at high concentrations weakly transported with P_Cs_/P_K_ values of 0.2 and 0.3 in native I_f_ or HCN4, respectively (D’Avanzo et al., 2009; Ho et al., 1994). At low sub-millimolar concentrations, Cs^+^ generates at negative voltages a strong voltage-dependent block (DiFrancesco, 1982; Moroni et al., 2000).

Here, we re-employ the MD simulation system with the open HCN4 PD (Saponaro et al., 2021a) to examine the interplay of the metal cations with increasing periodic numbers from the small Li^+^ to the largest Cs^+^ with the SF. This systematic study should provide further information on the dynamic selectivity mechanism in HCN channels in which the larger K^+^ ion appears to open up the pathway for Na^+^ conduction (Bauer et al., 2022). The mutual comparison between computational and experimental data should furthermore provide additional quality control for the cryoEM structure of the open HCN4 pore, which was obtained without applied voltage. Under these conditions, the channel is generally closed but was found in a presumably open state (Saponaro et al., 2021a). Hence, it is still debated if the pore region in the available open pore structure represents the time- and voltage-dependent activated open state of I_f_ and HCN channels, or the instantaneous open state (I_Inst_) of I_f_ and HCN (Accili, 2022). Since only the former open state is blocked by Cs^+^ (Proenza et al., 2002), the interaction of Cs^+^ with the open HCN4 pore will provide information on whether the available open channel structure reflects the pore in the time-dependent and voltage-activated state and provide us with some insight on the conduction mechanism in general.

## Materials and methods

### System preparation prior to simulations

MD simulations were performed as described previously (Bauer et al., 2022; Saponaro et al., 2021a) using the PD (L412–S523) of the cryoEM-solved apo HCN4 structure (PDB accession no. 7NP3) as the initial structure. This protein domain was inserted in a pre-equilibrated 1-palmitoyl-2-oleoyl-sn-glycero-3-phosphocholine (POPC) bilayer consisting of 130 molecules using CHARMM-GUI (Jo et al., 2008; Lee et al., 2016; Wu et al., 2014). Membrane-embedded PDs were solvated with 11,650 H_2_O molecules in a rectangular box (xyz: 80.137 Å × 80.137 Å × 102.024 Å). To yield an ion concentration of 900 mM while compensating the protein’s net charge of −8 e, 197 cations and 189 chloride anions were introduced by random replacement of H_2_O molecules. Different cation compositions were prepared for MD simulations, either consisting of one cation species exclusively, or of mixtures between Li^+^/Rb^+^/Cs^+^ and K^+^ in equal proportions. Additionally, 900 mM mixtures of Li^+^, Na^+^, and K^+^ were set up in equal ratios (Tables S2, S3, and S4).

Simulations were conducted with different arrangements of cation species within the SF and the cavity. Cations were placed in a central position between the four HCN4 monomers at an equal distance to the center of mass of the flanking amino acids.

Two-ion states were set up with cations in p23 (G481) and p34 (C479), with a separating H_2_O molecule in between in the S3 (I480) site and an additional cation in the cavity below p34 (between L478 and Y507). These ion configurations were chosen in concordance with stable ion placements in the two-ion state determined in Saponaro et al. (2021a). For Rb^+^ and Cs^+^, additional configurations with one cation in the vestibule above p23, the second in S3, and the third in the cavity were set up.

### Equilibration regime and MD simulations

The whole MD simulation procedure was performed with GROMACS 2019.6 (Abraham et al., 2015; Berendsen et al., 1995; Pall et al., 2015). The Amber99sb*-ILDN force field was used in combination with the TIP3P water model, Berger lipid-derived parameters for POPC and the TIP3P-optimized ion parameter set derived by Cheatham and Joung was used (Berger et al., 1997; Best and Hummer, 2009; Jorgensen et al., 1983; Joung and Cheatham, 2008; Lindorff-Larsen et al., 2010). Van der Waals interactions were cut off for distances above 1 nm, whereas Coulomb interactions were treated with the particle-mesh Ewald method with a 1 nm real space cutoff. The V-rescale thermostat was used to keep the temperature at 310 K (Bussi et al., 2007). The pressure was held constant at 1 bar using the Berendsen and the Parinello-Rahman barostat for equilibration and production simulations respectively (Berendsen et al., 1984; Parrinello and Rahman, 1981). To enable simulations with an integration time step of 4 fs, all covalent bonds were restrained using the LINCS algorithm, and hydrogen atoms were represented as virtual sites (Feenstra et al., 1999; Hess et al., 1997).

Following the system preparation, 2,000 steps of energy minimization were conducted with the steepest-descent integrator and a step-width of 0.1 Å. This was followed by equilibration in the NVT ensemble for 100 ps with restrained protein backbone and sidechains (F_c_ = 1,000 kJ mol^−1^ nm^−2^), followed by 20 ns of restrained simulations in the NPT ensemble. Restraints were gradually lifted over successive NPT equilibration steps (Table S1). Production simulations were run for 500 ns or 1 μs with an applied electric field (between −150 and −700 mV). To observe permeations in MD simulations in a reasonable simulation time, high voltages (−500/−700 mV) were applied to compensate for the low unitary conductance of HCN4.

Only the membrane-embedded isolated open pore module of HCN4 was considered in MD simulations to reduce computational demand. To compensate missing voltage sensor and cytosolic domains and keep the pore in its open state, restraints were applied to residues A415, I511, T515, and G519 using PLUMED v. 2.7.0 (Kopec et al., 2018; Tribello et al., 2014). H_2_O molecules in simulations with the isolated pore were counted within a 5 Å cut-off-range of residues 507–511 to monitor whether the helix bundles at the lower cavity remain in an open state. These values were compared with MD simulations of the whole channel protein in the apo-open and holo-closed state (Bauer, 2021). Similar H_2_O counts for the PD and the apo-open channel confirm that the restraints described above effectively retain the PD in its open state (Fig. S7, B and C).

### Analysis of trajectories and filtering

The resulting MD trajectories were analyzed using GROMACS 2019.6 and 2023 as well as the Biotite python library for bioinformatics and MD analysis in versions 0.32–0.35 (Berendsen et al., 1995; Kunzmann and Hamacher, 2018). NumPy and Pandas were additionally used for analysis, Matplotlib and seaborn for data visualization (Harris et al., 2020; Hunter, 2007; McKinney, 2010; Waskom, 2021).

To compute the density of cations in the channel pore, cylindrical volume elements were created using the initial pore structure (Fig. S8): Cylinder centers were aligned to the center of mass of carbonyl oxygens in the selectivity filter; the height of each cylinder corresponds to the distance between C479 carbonyl oxygens and those found in I480. The minimal xy distance between C_α_ atoms of flanking amino acids and the corresponding cylinder center in each height interval was adopted as the radius. With four additional cylinders added above and below the membrane in the bulk solution, a total number of 22 cylinders was generated. To correct for lateral diffusions of the whole pore during MD simulations, cylinders were recentered on the current center of mass of SF carbonyl oxygens.

For the computation of site-specific dwell times, a Butterworth lowpass filter implemented in the SciPy library v. 1.9.3 was applied to trajectories to reduce fluctuations and allow for an automated assignment of cations to binding sites according to their z coordinates (Virtanen et al., 2020). Here, a cutoff frequency of 10 ns^−1^ was chosen in combination with a third-order Butterworth filter.

Site-specific hydration numbers were computed using cation-specific cutoffs of 2.74, 3.21, 3.55, 3.8, and 4.1 Å for Li^+^, Na^+^, K^+^, Rb^+^, and Cs^+^, respectively (Lamoureux and Roux, 2006).

To verify sufficient equilibration, protein backbone root-mean-square deviations of all individual trajectories were monitored (not shown). In all analyses for which data of multiple trajectories were aggregated, the first 50 ns were discarded accordingly.

Four additional simulations with the HCN4 pore and Na^+^ as well as mixed Na^+^/K^+^ drawn from Saponaro et al. (2021a) were additionally included in aggregated analyses shown in Figs. 9 and 10 (Table S5 and Fig. S6).

Trajectory frames were visualized as molecular structures using the Biotite extension Ammolite v. 0.8 and PyMOL Open Source v. 2.5.0 (Schrödinger, Inc., 2017).

### Electrophysiological measurements

HEK293 cells stably expressing HCN2 (Stieber et al, 2005) were generously provided by Dr. J. Stieber (University Erlangen, Erlangen, Germany). Cells were grown at 37°C in a humidified 95% air/5% CO_2_ incubator in Dulbecco’s modified Eagle medium (DMEM; Gibco) supplemented with 10% v/v heat-inactivated fetal bovine serum, 100 U/ml penicillin G, 100 μg/ml streptomycin sulfate, and 2 mM L-glutamine (all from Invitrogen). After reaching ∼80% confluence, cells were dispersed by accutase treatment and after washing in the incubation medium seeded on 15-mm cover slips. HCN2 currents in single HEK293 cells were measured in the whole-cell configuration with an EPC-9 amplifier (HEKA Elektronik) at room temperature (20–25°C). Pipettes were pulled from borosilicate capillaries (DWK Life Sciences) on a microelectrode puller (PP-830; Narishige Group) resulting in pipettes with 2–5 MΩ resistances. The capillaries were coated at tapper with Sigmacote (Merck KgaA) and baked after pulling at 65°C for 45 min.

The pipette solution contained (in mM) 8 NaCl, 104 KCl, 0.1 CaCl_2_, 5 4-(2-hydroxyethyl)-1-piperazineethanesulfonic acid (HEPES)/KOH pH 7.2, 1 ethylene glycol-bis(β-aminoethyl ether)-N,N,N′,N′-tetraacetic acid (EGTA), and 2 MgATP. The external bath solution contained (in mM) 110 NaCl, 30 KCl 1.8 CaCl_2_. 0.5 MgCl_2_, and 5 HEPES/KOH, pH 7.4. For the test of selectivity, NaCl and KCl were replaced by 140 mM CsCl or LiCl. Osmolarity was adjusted to 240 and 280 mOsmol/kg in pipette and bath, respectively, with D-mannitol.

Currents were recorded in response to a two-sweep voltage protocol. In the first sweep, the cell was clamped from resting voltage for 712 ms to −40 mV followed by a fast voltage ramp to −250 mV (12 mV/ms). After allowing the cell to relax at the resting voltage for 5 s, it was clamped in a second sweep first for 720 ms to −130 mV followed by a fast ramp (12 mV/ms) to −250 mV. Currents were filtered at 10 kHz using a low pass Bessel filter and sampled at 20 kHz without leak current subtraction. Data was collected with PatchMaster (HEKA Elektronik) and analyzed with FitMaster (HEKA Elektronik).

### Fluorescence size exclusion chromatography-based thermostability

GFP-tagged HCN4 protein, transiently expressed in HEK293F cells (Cat# R79007; Thermo Fisher Scientific) and purified in LMNG-CHS detergent mixture, as detailed in Saponaro et al. (2021a), was subjected to the fluorescence size exclusion chromatography-based thermostability (FSEC-TS) assay following the procedure detailed in Saponaro et al. (2021b). 50 mM CsCl was added, when needed, prior to the FSEC-TS.

### Online supplemental material

**Figure 2.**
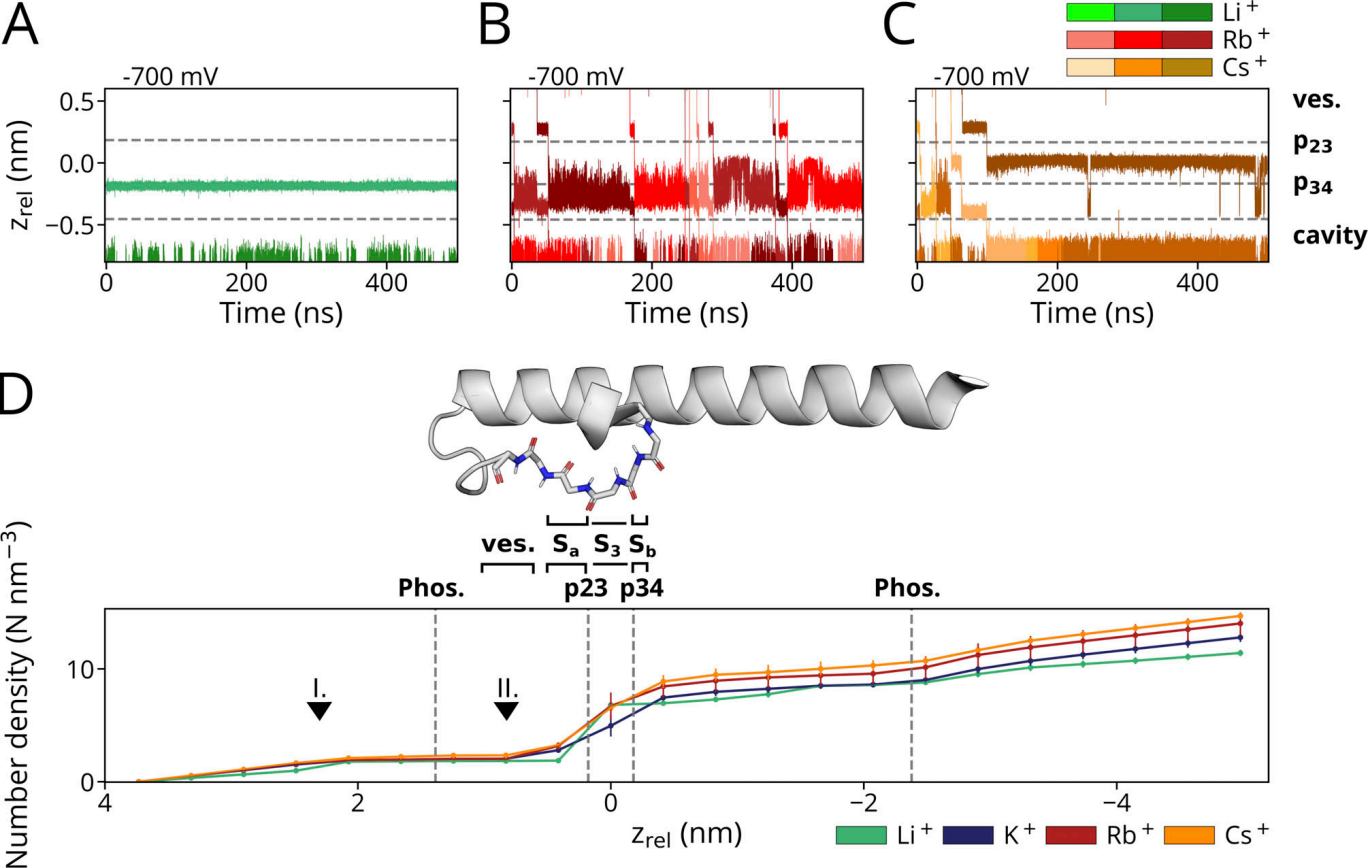
**Characteristic ion trajectories depicting primary binding sites of Li**^**+**^**, Rb**^**+**^**, and Cs**^**+**^ **obtained from simulations conducted at −700 mV in solutions with a single cation species. (A)** With prolonged presence of Li^+^, p34 was identified as a single binding site within the SF. Permeation of Li^+^ through p23 was only observed in a single instance. **(B)** Similar to K^+^, Sa (encompassing p23) and Sb/Sc (proximate to p34) were identified as binding sites within the SF for Rb^+^. Intermediate fluctuations between Sb and S3 in the single-ion state are observable at higher voltages, while prolonged binding to S3 occurred at lower voltages. **(C)** Cs^+^ is predominantly found as a single cation in the S3 site in all simulations. Both Rb^+^ as well as Cs^+^ are found in a {Sa, Sc} configuration during conduction events. **(D)** Empirical CDF of computed numerical densities (in counts/nm^3^) for Li^+^, Rb^+^, and Cs^+^ conducted at −700 mV (*n* = 4, *n* = 1, and *n* = 2, respectively), including simulations depicted in A–C and of K^+^ (*n* = 4; pure KCl), and Rb^+^ (*n* = 3, pure RbCl) at −500 mV along the z-axis of the simulation box. The mean z positions of p23, p34, and the POPC-headgroup phosphor atoms (Phos.) are marked as dashed lines. Arrowheads denote reference z_rel_ positions along the pore axis: Unequal count densities are observed for Li^+^ compared to the other cations (I.), whereas similar number densities are observed in the vestibule above the SF (II.). For reference, the channel pore-facing helix and the SF of a single HCN4 monomer are shown above the subplot. The vestibule (ves.) and SF binding sites Sa, S3, and Sb are separately outlined.

**Figure S1.**
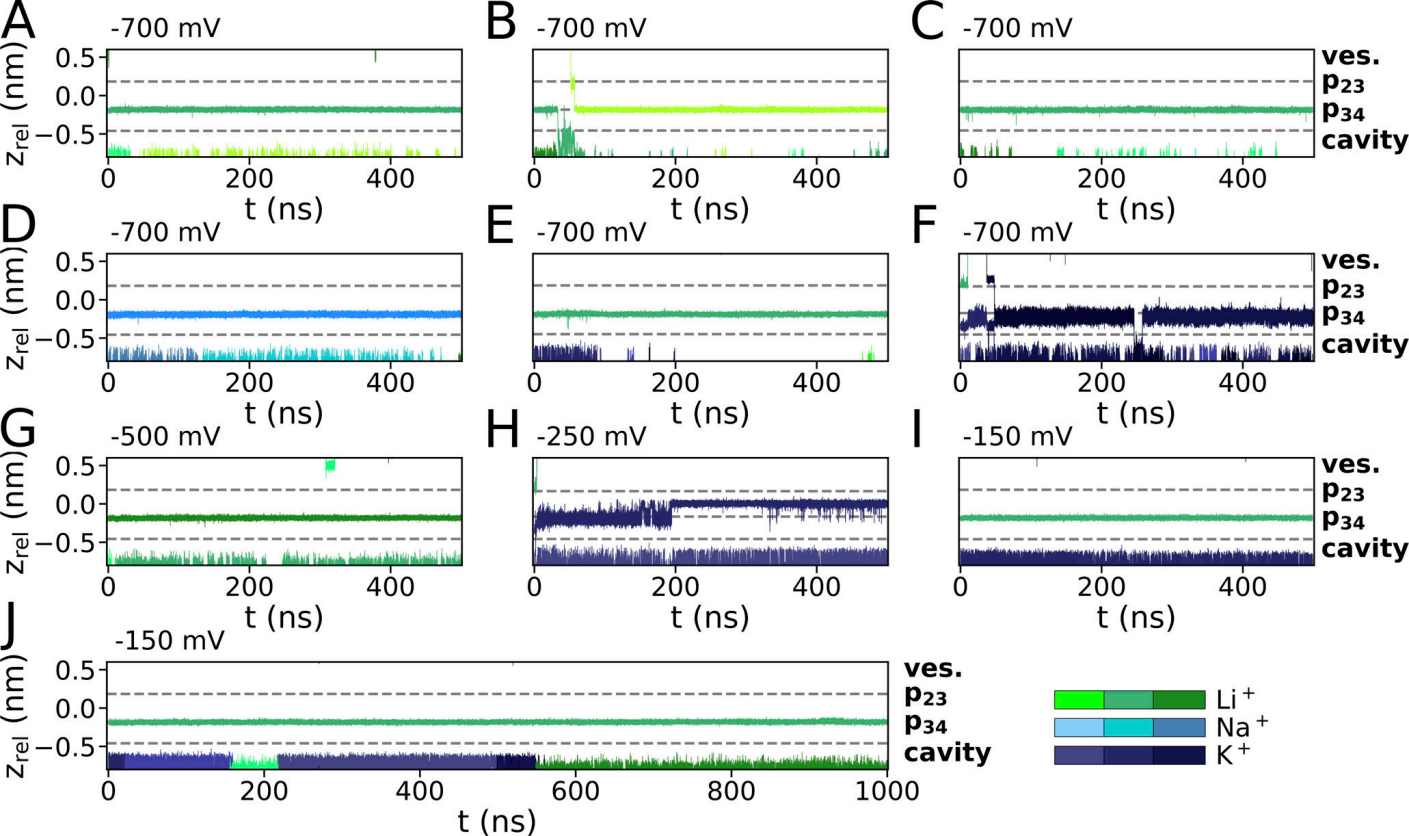
**Ion trajectories for simulations with Li**^**+**^**/Na**^**+**^ **and K**^**+**^ **(900 mM) at various membrane voltages. (A–F)** Hyperpolarizing voltages of −700 mV were applied for pure LiCl with Li^+^ preplaced in p34. **(D–F)** Simulations were conducted at the same voltage and mixed-cationic sets for (D) LiCl/NaCl with Na^+^ preplaced in p34, (E) LiCl/KCl with Li^+^ preplaced in S3, (F) LiCl/KCl with Li^+^ and K^+^ preplaced in p23 and p34, respectively. **(G–J)** Additional simulations at lower hyperpolarized membrane potentials with initial placement (G) of Li^+^ in p34 and within the cavity, (H) Li^+^ in p23 and K^+^ in p34 and (I and J) Li^+^ in p34 were conducted with −500, −250, and −150 mV, respectively.

**Figure S2.**
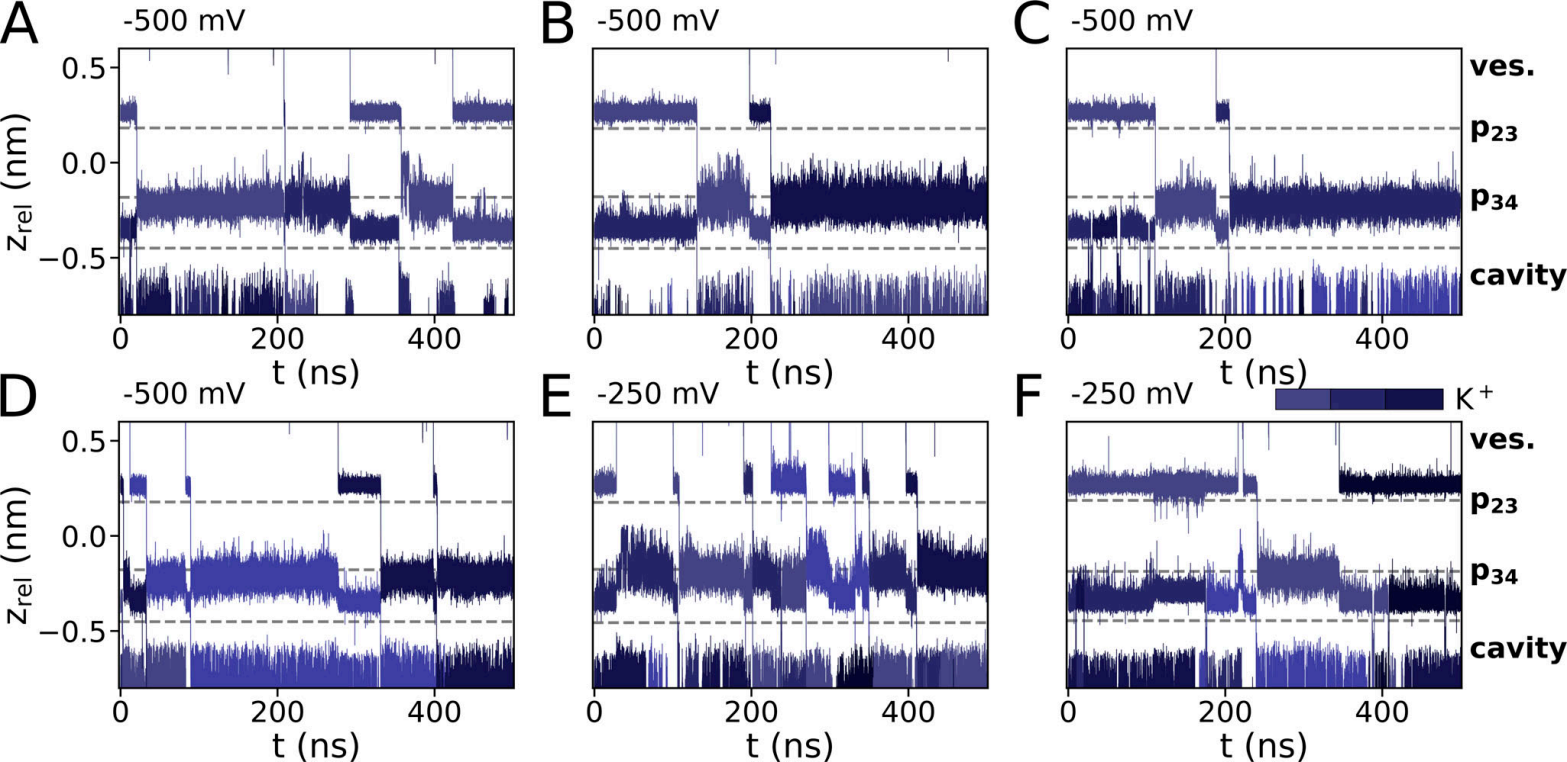
**Ion trajectories for simulations with KCl (900 mM), with cations preplaced in p23 and p34.** Simulations were run with applied voltages of (A–D) −500 mV and (E and F) −250 mV.

**Figure S3.**
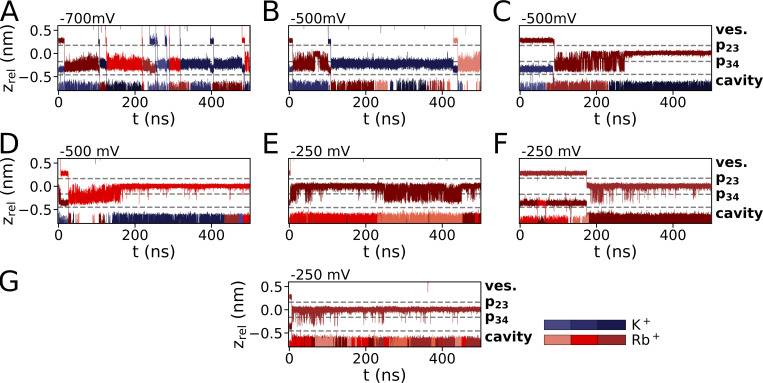
**Ion trajectories for simulations with K**^**+**^**/Rb**^**+**^ **and pure Rb**^**+**^**. (A–D)** Mixed cationic KCl/RbCl simulations with Rb^+^ preplaced in p23 and K^+^ in p34 were conducted at (A) −700 mV and (B and C) −500 mV. A simulation with a mixed cationic solution at −500 mV and preplacement of Rb^+^ in p23 and S3 is shown in D. **(E–G)** Simulations conducted for RbCl, with initial Rb^+^ placement in p23 and p34 at −250 mV are shown in the second row.

**Figure S4.**
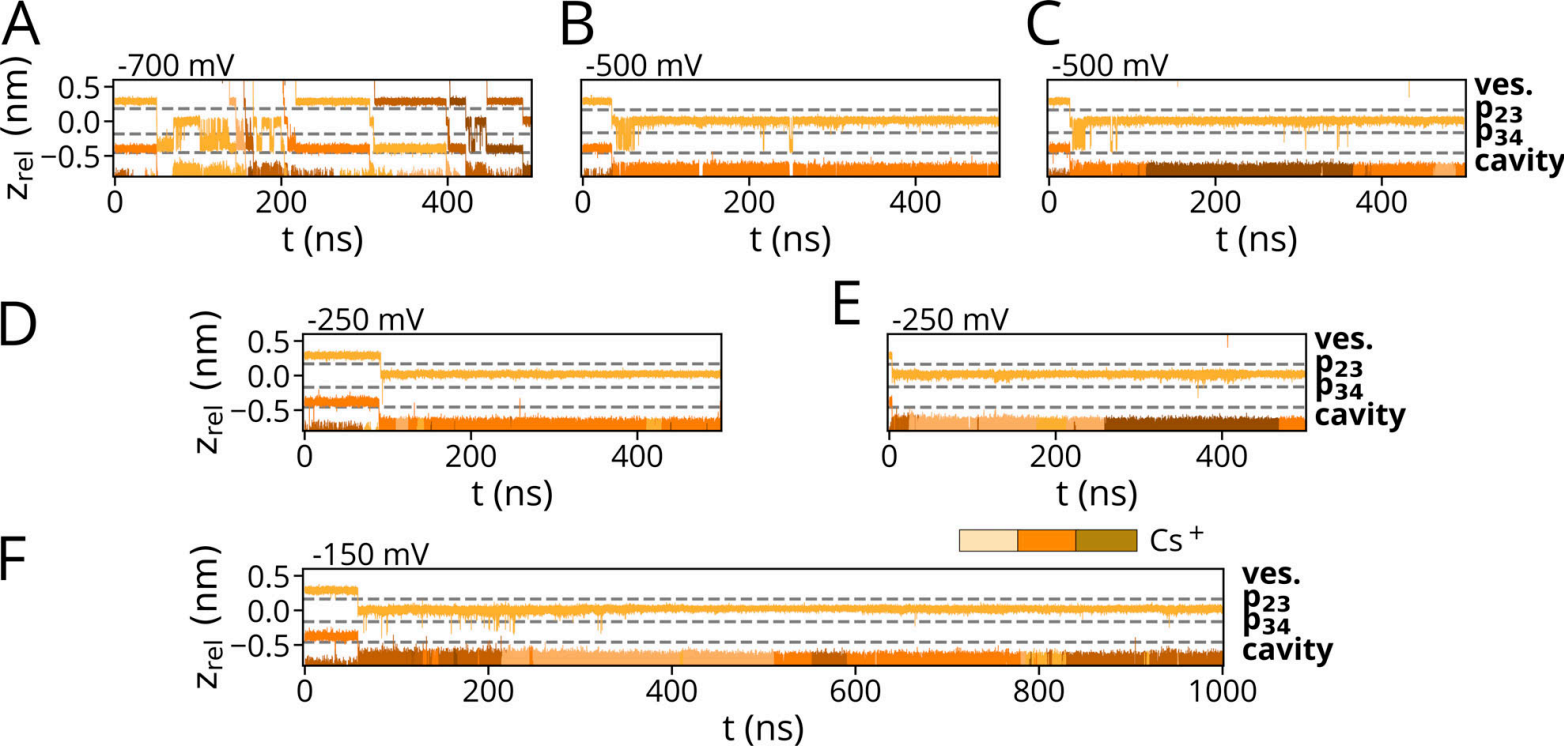
**Ion trajectories for simulations with pure CsCl. (A–F)** For simulations with CsCl (900 mM) with Cs^+^ preplaced in p23 and p34 from simulations conducted at −700 mV (A), −500 mV (B and C), and −250 mV (D and E). A simulation conducted at −150 mV and a cation concentration of 500 mM is depicted in F.

**Figure S5.**
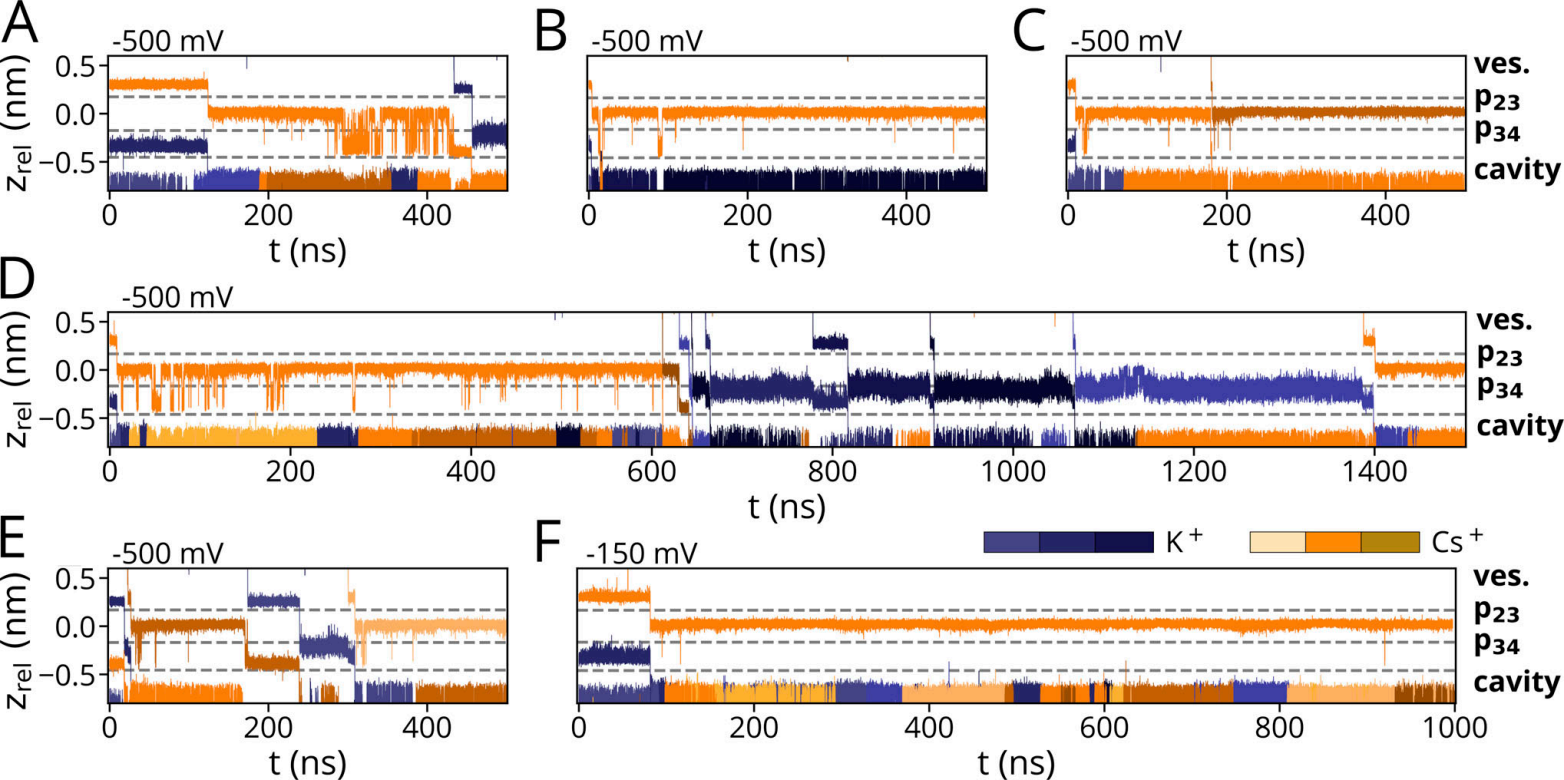
**Ion trajectories for mixed-cationic simulations with Cs**^**+**^ **and K**^**+**^ **preplaced in p23 and p34, respectively. (A–F)** Simulations were conducted at −500 mV with a 900 mM cation concentration (A–E) and at −150 mV/500 mM (F).

**Figure S6.**
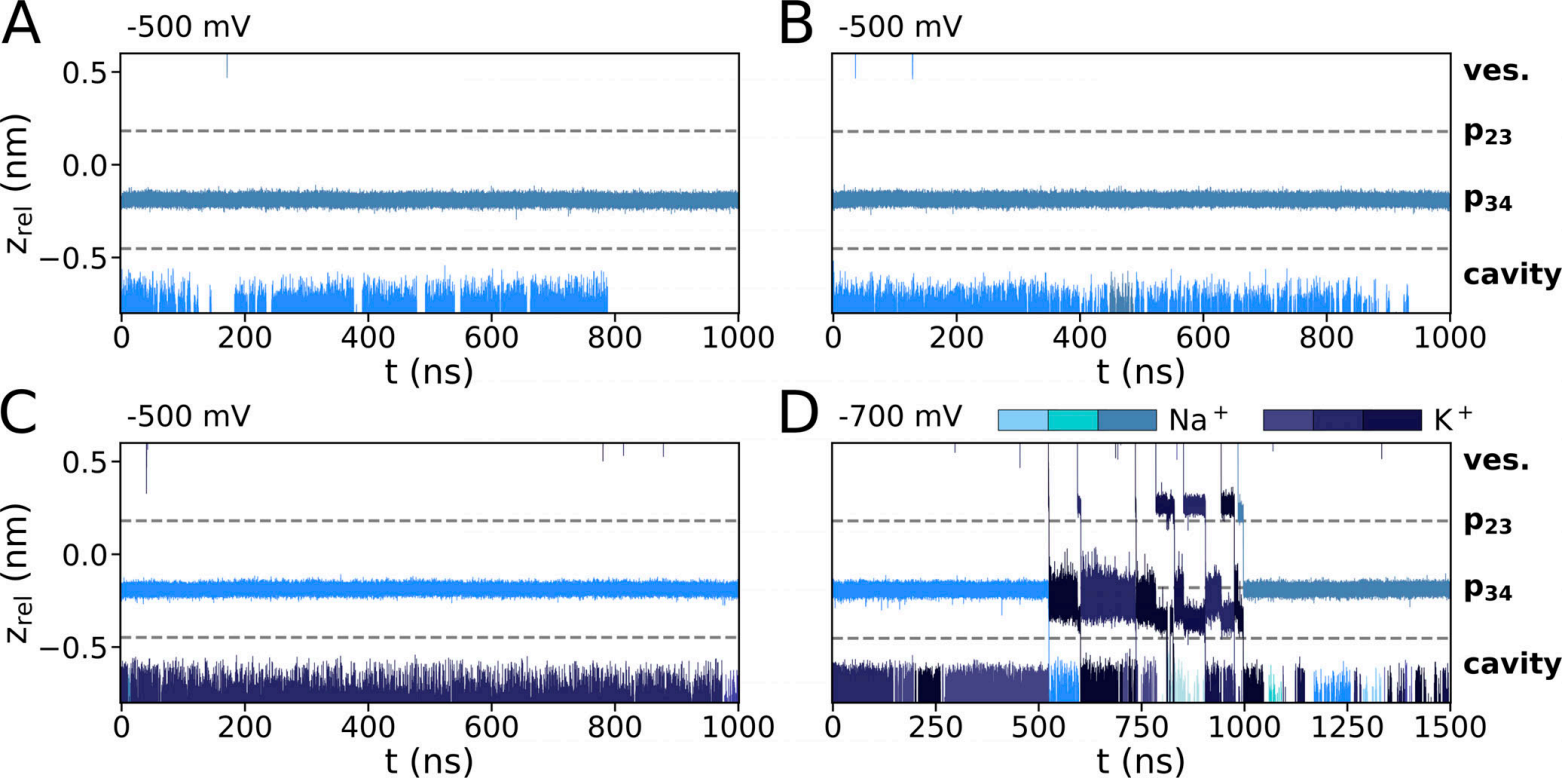
**Ion trajectories for mixed-cationic simulations with Na**^**+**^ **and K**^**+**^ **from the previously published dataset (**Saponaro et al., 2021a**). (A–D)** Simulations were conducted in 900 mM Na^+^ at −500 mV (A and B) as well as 900 mM mixed Na^+^/K^+^ (1:1) at (C) −500 mV and (D) −700 mV.

**Figure S7.**
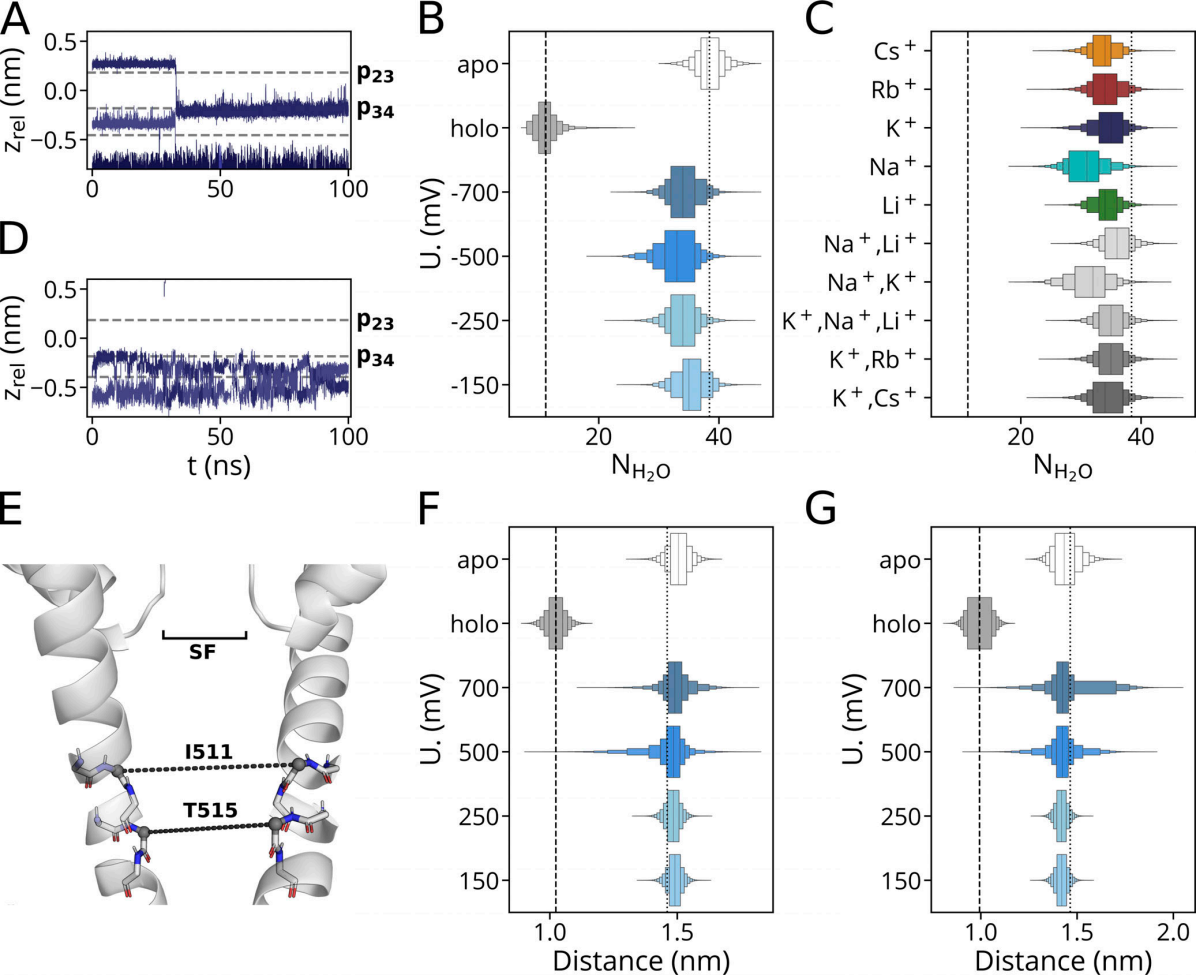
**Comparison of critical parameters from simulations with entire HCN4 proteins and isolated channel pore. (A–D, F, and G)** In simulations with entire channel protein in 150 mM K^+^ (Bauer, 2021), potassium ion transitions can be observed in the apo-open (A) but not in the holo-closed form (B). Pore hydration in the cavity (C and D) and critical helix distances (F and G) at the entrance to the cavity from these simulations were taken as reference for the open and closed state of the channel, respectively. Both parameters were compared with the respective values from simulations with the isolated pore under various experimental conditions. Data in C show hydration as the number of water molecules (N_H2O_) in the cavity of the entire channel protein in the apo-open (white) and holo-closed (gray) state as in A and B compared with simulation results with the isolated pore at different voltages (blue shades). Data in D show the analysis of the same parameter as in C from simulations with the isolated pore in pure or mixed cation solutions depicted on the left. Dotted and dashed lines respectively indicate mean values from the simulation of the entire protein in the open and closed states as a reference in both C and D. **(E–G)** Distances between C_α_ atoms of I511 and T515 in opposite subunits (E) depicted in F, are critical markers for the open or closed conformation of the channel. The distributions of the respective distances from simulations of the entire protein in apo-open or holo-closed form as well as the respective distances from simulations with the isolated pore run at different voltages are shown for I511 (F) and T515 (G). All simulations with the isolated open pore confirm that it remains under different simulations conditions in the open state. Only trajectory frames corresponding to t_sim_ ≥ 50 ns were included in these analyses.

**Figure S8.**
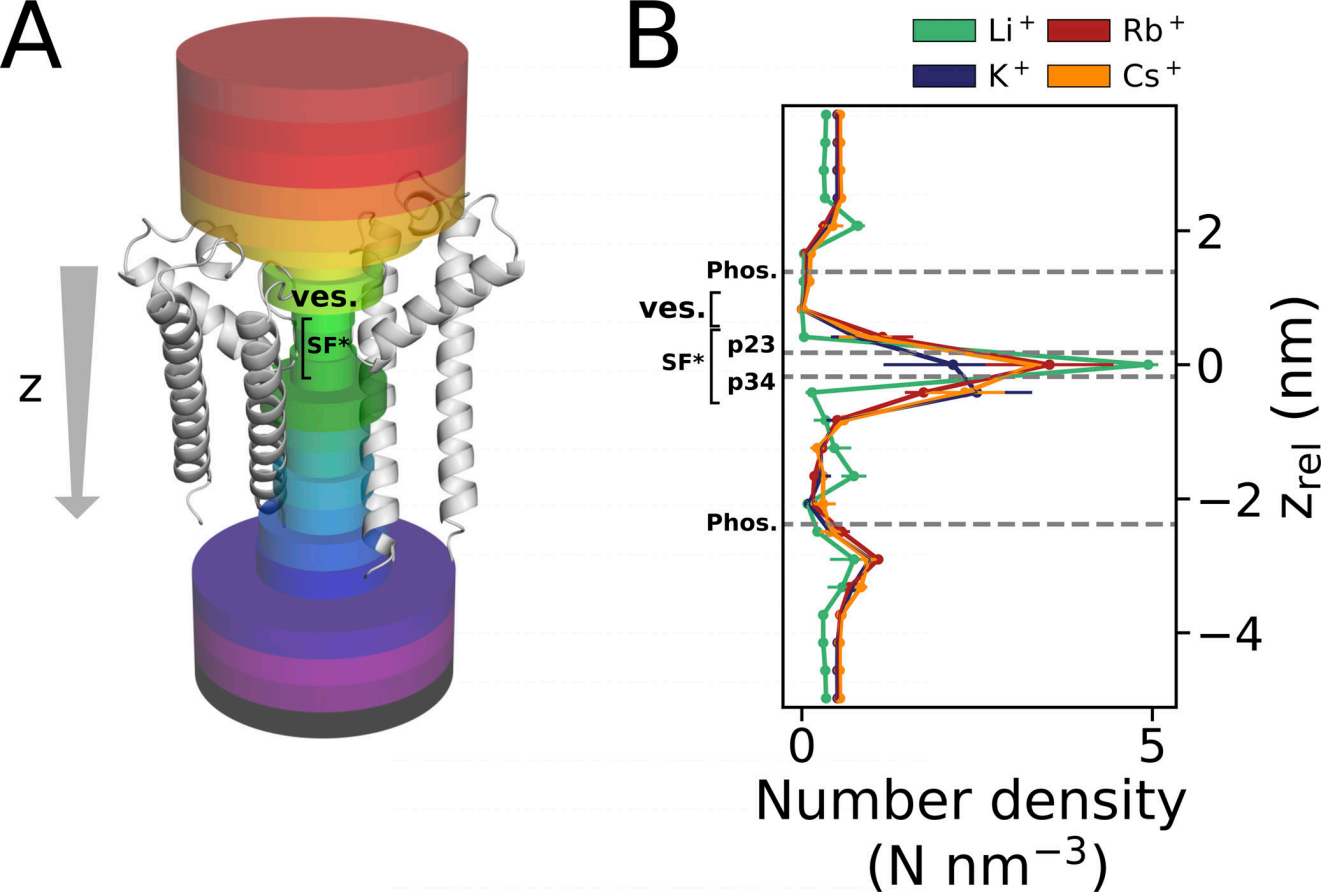
**Counting the number of cations in the pore. (A and B)** Schematic representation of cylinder-shaped zones for counting the number of cations within the pore (A) and count density distribution underlying CDF plot in Fig. 2 D (B). The three zones encompassing Sa (p23), Sb (p34), as well as S3 and the zone corresponding to the vestibule, are separately marked as SF* and ves., respectively.

Additional ion trajectories for different cation compositions and voltages are depicted in Figs. S1, S2, S3, S4, S5, and S6. Ion trajectories of K^+^ ions from simulations with the entire HCN4 channel in apo-open and holo-closed state are reported in Fig. S7. Mean pore hydration as well as distances at the cytosolic entrance to the cavity from simulations with the entire channel are compared with respective values from simulations with the isolated PD in the open configuration. A schematic representation of the geometrical shapes used to compute the cumulative distribution function (CDF) in Fig. 2 D and the corresponding number density distribution are depicted in Fig. S8. Table S1 lists the equilibration steps performed prior to production simulations with applied protein restraints. All simulations are listed in Tables S2, S3, and S4; simulations from an older dataset were reanalyzed and listed in Table S5. Literature values for cationic radii and hydration numbers are listed in Table S6.

## Results

### Cation binding sites and ion/protein interaction patterns

To examine the interaction of different cations with the SF in the open HCN4 channel pore, we performed MD simulations of the membrane-embedded PD in solutions containing the cation of interest together with Cl^−^ as the anion. In complementary experiments, we used mixed ion solutions. Because of the notoriously low unitary conductance of HCN channels, all simulations were first performed with high salt concentration (900 mM) and at high negative voltages (−700 and −500 mV); this increased the chance of detecting ion transitions during reasonable simulation run times. To rule out the eventuality that results are artifacts of high voltage, selected simulations were also repeated at lower voltages (−250 and −150 mV).

### Li^+^ is not entering the selectivity filter

In three out of four 500-ns simulations conducted at −700 mV in 900 mM LiCl solution with a Li^+^ ion preplaced in Sb/p34, we observed no transitions (Fig. 2 A; and Fig. S1, A and C). Only in a single instance, a peculiar Li^+^ permeation event was observed in which a cation was passing through an otherwise empty selectivity filter, without direct replacement of a cation in S3 or Sb (Fig. S1 B). Like in simulations in pure NaCl solutions (Saponaro et al., 2021a), the Li^+^ ion was permanently kept in binding site Sb in the carbonyl oxygen plane p34, i.e., the position also preferred by Na^+^. The same prolonged binding to Sb was observed in a 1-µs long reference simulation at −150 mV, underscoring that the immobility of the Li^+^ ion is not an artifact of the high voltage (Fig. S1 J).

**Figure 3.**
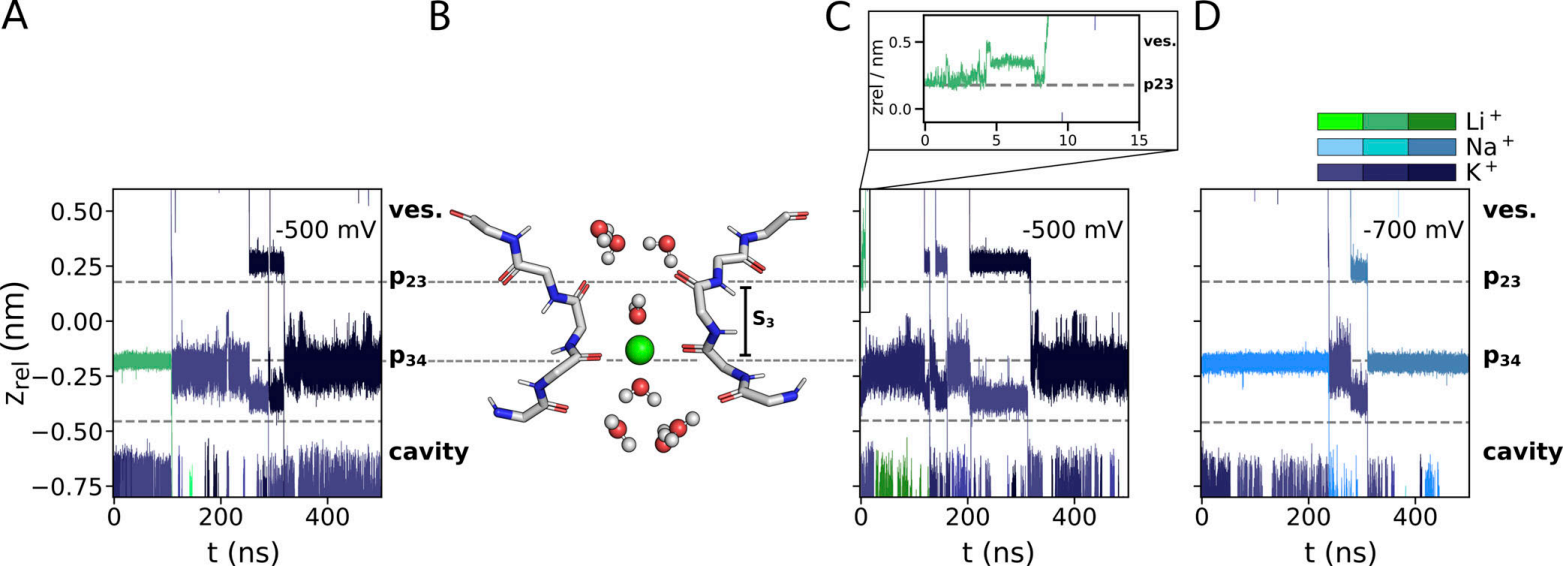
**Ion traces depicting the movement of Li**^**+**^**, Na**^**+**^**, and K**^**+**^ **(green, light, and dark blue, respectively) along the main channel axis from simulations with Li**^**+**^ **at varying initial positions, voltages, and ion compositions. (A and B)** −500 mV with Li^+^/K^+^ and preplacement of Li^+^ in p34 with B as snapshot of a trajectory with Li^+^ residing in binding site Sb. **(C)** −500 mV with Li^+^/Na^+^ and Li^+^ preplaced in p23. The rapid diffusion of Li^+^ into the vestibule is depicted in the inset with higher temporal resolution. **(D)** −700 mV with Li^+^/Na^+^/K^+^ and Na^+^ preplaced in p34.

To test if the permeant K^+^ ion has, like Na^+^, a stimulating effect on Li^+^ transport, simulations were repeated in mixed Li^+^/K^+^ solutions with a K^+^ ion preplaced into Sa/p23 and Li^+^ in Sb/p34 (Fig. 3 A). With this starting configuration, we observed no permeation of Li^+^ at potentials between −700 and −150 mV (Fig. 2 A and Fig. S1 I). Here, we define permeations as transitions of a cation from Sa to the cavity.

Further simulations were conducted by placing Li^+^ in different starting conditions. In none of these cases did we observe Li^+^ crossing the p23 site (Fig. 3 C; and Fig. S1, F and H). An interesting case in this context is a simulation with a mixed Li^+^/K^+^ solution in which Li^+^ was preplaced at the height of p23 (Fig. 3 C). Li^+^ rapidly diffused out of this site into the vestibule and subsequently into the external bulk. This phenomenon which was also seen in additional simulations where Li^+^ was preplaced in the externally facing side of the SF (Fig. S1, C and E) tentatively suggests that this site is not a potential binding site for the small cation. This conclusion has to be taken with caution since Li^+^ left this site already during the first few nanoseconds of MD simulations, i.e., at a time in which the system is not yet fully equilibrated.

In simulations with mixed solutions, we found that Na^+^ and K^+^ were still able to enter the selectivity filter after Li^+^ had left its position in Sa. An example is shown in Fig. 3 D, where K^+^ ions entered and fully passed the filter after Li^+^ had left Sa. The results of these experiments predict that Li^+^ in the external medium is not suppressing K^+^ currents in HCN4. The stable binding of Li^+^ to Sb, which was observed in Fig. 2 A, is presumably not relevant under physiological conditions: it is unlikely that Li^+^ reaches this site from the external bulk medium.

A comparison between simulations in Li^+^ versus Na^+^ solutions shows that the two ions have different behavior. The latter cation remains like Li^+^ bound to Sb in the absence of K^+^ but enters and passes the filter in the presence of K^+^ in the bulk solution (Fig. 3 D). The different behavior of Li^+^ and Na^+^ predicts that the HCN4 channel is not conducting Li^+^ because the small cation is not able to enter the SF from the external bulk solution for binding to Sb. In this context, it is interesting to mention that Li^+^ has a higher propensity than other cations for binding to charged lipid membranes, and Li^+^ as well as Na^+^ are found in higher numbers proximate to phospholipid bilayers compared with larger alkali metal cations (Cordomí et al., 2008; Klasczyk et al., 2010; Kruczek et al., 2017; Maity et al., 2016). Hence, the failure of Li^+^ entry to the HCN4 filter could be augmented by the fact that the membrane creates a sink for Li^+^ ions preventing them from approaching the entry vestibule of the channel to the same extent as K^+^. To examine this hypothesis, we calculated the CDF for all cations of interest for the transfer from the bulk solution into the HCN4 channel pore. The data in Fig. 2 D show that the number of mobile Li^+^ ions in the external solution above the bilayer is indeed partially lower than that of the other three cations. While the local concentration of Li^+^ ions increases more steeply than that of K^+^/Rb^+^/Cs^+^ right above the aqueous/lipid interface (Fig. 2 D, arrow I.), all cations show only slight increases in density along the channel pore between membrane and SF. All cations exhibit similar local densities in the vestibule above the Sa site along the z-axis (Fig. 2 D, arrow II.). In the pore above the SF, no steep increases in the CDF are visible that would hint specific cation–binding sites along the main pore axis. We therefore conclude that the absence of Li^+^ conductance in the present simulations is likely the result of local interactions of the small cation with the SF. Phospholipids are not scavenging, thereby drastically reducing the number of available Li^+^ ions in the vicinity of the filter in the simulation.

Taken together, the results of our simulations with the small Li^+^ cation are in good agreement with experimental data. Like in the simulations, electrophysiological recordings have also shown that neither HCN channels nor the I_f_/I_h_ currents are conducting Li^+^ to any appreciable degree (D’Avanzo et al., 2009; Ho et al., 1994; Wollmuth and Hille, 1992). In further experiments, it was also shown that the small cation is not an efficient blocker of the I_f_ conductance (Ho et al., 1994).

### Rb^+^ conducts in a different manner from K^+^

In simulations in 900 mM RbCl, we repeatedly observed full transitions of Rb^+^ ions through the entire PD (Fig. 1 B). In the case of Fig. 1 B, eight Rb^+^ ions are fully passing the pore during a 500-ns long simulation. Frequent transition events of Rb^+^ were also observed in mixed Rb^+^/K^+^ solutions, even at moderate negative voltages.

**Figure 4.**
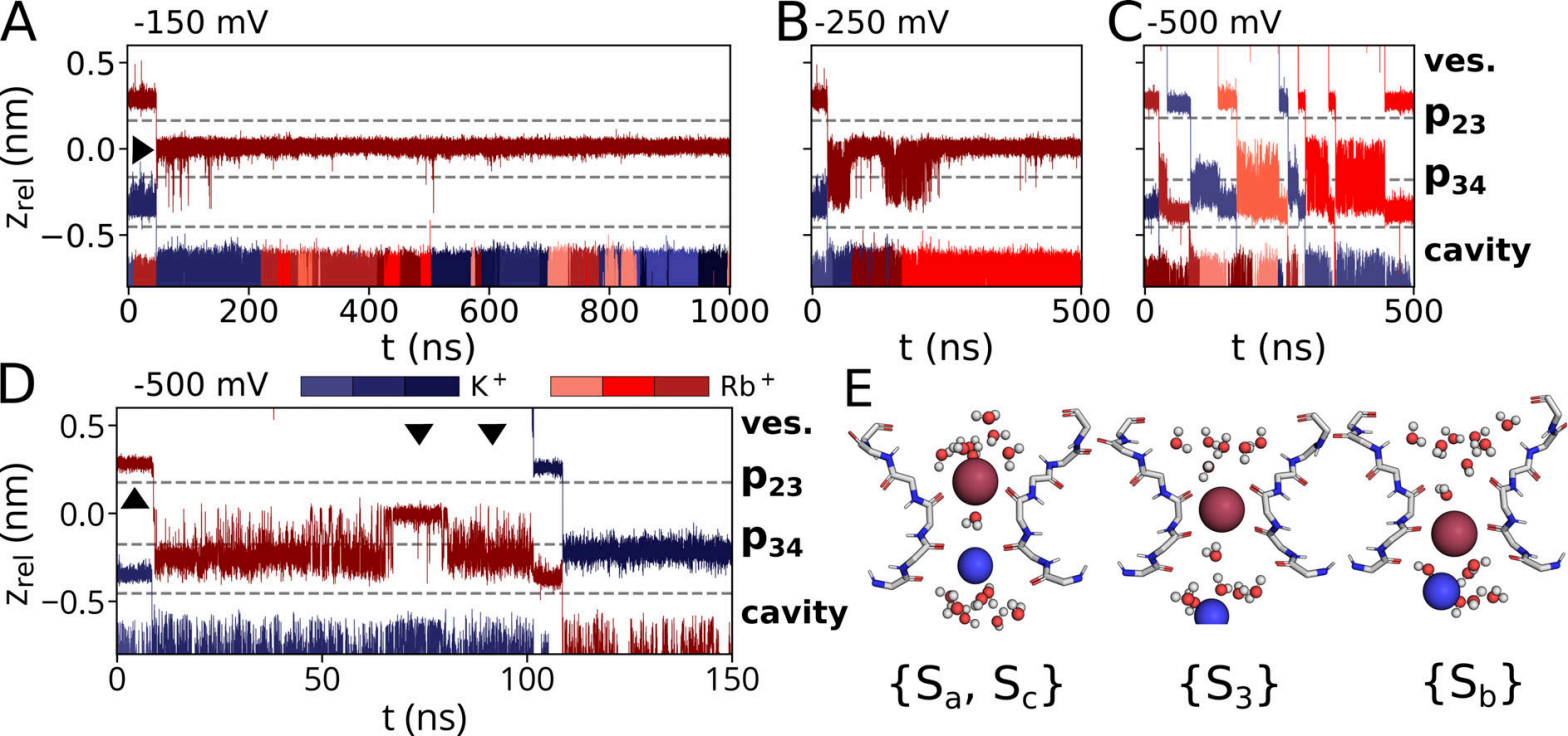
**Preferred binding sites of Rb**^**+**^ **within the SF. (A–C)** Conduction trajectory for Rb^+^ along the SF axis from simulations in mixed-ionic solutions with K^+^, at different voltages, and for different initial cation placements within the SF: Rb^+^ is preplaced in p23 and K^+^ in p34 at simulations with an applied voltage of (A) −150 mV, (B) −500 mV, and (C) −700 mV. The arrowhead in A indicates a direct Sa–S3 transfer. **(D and E)** Details of conduction mechanisms at −500 mV are shown in D with corresponding snapshots (E) taken at times indicated by arrowheads in D.

Fig. 4 shows trajectories of 500-ns long simulations with mixed Rb^+^/K^+^ solution in which Rb^+^ was preplaced in Sa in proximity to p23 at the start of the simulation. In this condition, frequent transitions of either K^+^ or Rb^+^ were observed at −500 mV (Fig. 4 C) but not at lower voltages. The transition events are too few for a robust statistical comparison between the two ions. But we can assume from the simulation data at −500/−700 mV that the channel is conducting both cations with a similar rate (Fig. 4 C; and Fig. S3, A and B). These results are in good agreement with experimental data showing that Rb^+^ is well transported by the I_f_ current and the underlying HCN channels (D’Avanzo et al., 2009; DiFrancesco, 1982; Ho et al., 1994).

A detailed comparison of ion transitions in either pure Rb^+^ or K^+^ solutions, or from mixed Rb^+^/K^+^ solutions, shows that both cations are permeating the filter in distinctly different manners (Fig. 2 B; Fig. 4, A, B, and D; and Figs. S2 and S3). The general behavior of Rb^+^ is independent of the presence of K^+^ because the same Rb^+^ transition pattern was observed under both pure Rb^+^ and mixed Rb^+^/K^+^ conditions: like in the case of K^+^ solutions, the filter can accommodate either one or two ions (Fig. 2 B and Fig. 4 C). Also, like K^+^, Rb^+^ ions are primarily kept in {Sa, Sc} two-ion states (Fig. 4 C). With only one ion in the pore, Rb^+^ visits the S3 site from which it oscillates over long periods with a high frequency back and forth between S3 and Sb; from there, it finally transitions into a {S3} single-ion state (Fig. 4 B) or leaves the filter via Sc into the cavity on the arrival of a new ion from the external bulk (Fig. 4, C and D). The sequence of events in Fig. 4 D nicely visualizes the difference between the two cations in the same simulation: Rb^+^, but not K^+^, stays for an appreciable time in S3. Also, while the K^+^ ion has a strong bias for Sb over S3, the Rb^+^ ion oscillates for extensive periods back and forth between both sites. Simulations at different voltages, however, underpin that these oscillating transitions of Rb^+^ between S3 and p34 are voltage-dependent: lower membrane potentials seem to further stabilize the single-ion configuration with Rb^+^ in S3 (Fig. 4, A and B; and Fig. S3). Correspondingly, an alternative conduction pattern with a direct transfer of the upper cation into S3 after a {Sa, Sc} two-ion state is observed in a simulation conducted at −150 mV (Fig. 4 A, arrowhead) whereas both Sa–Sb transitions and Sa–S3 transitions were observed at −250 mV (Fig. 4 B; and Fig. S3, F and G).

### Cs^+^ is binding to the canonical S3 site

**Figure 5.**
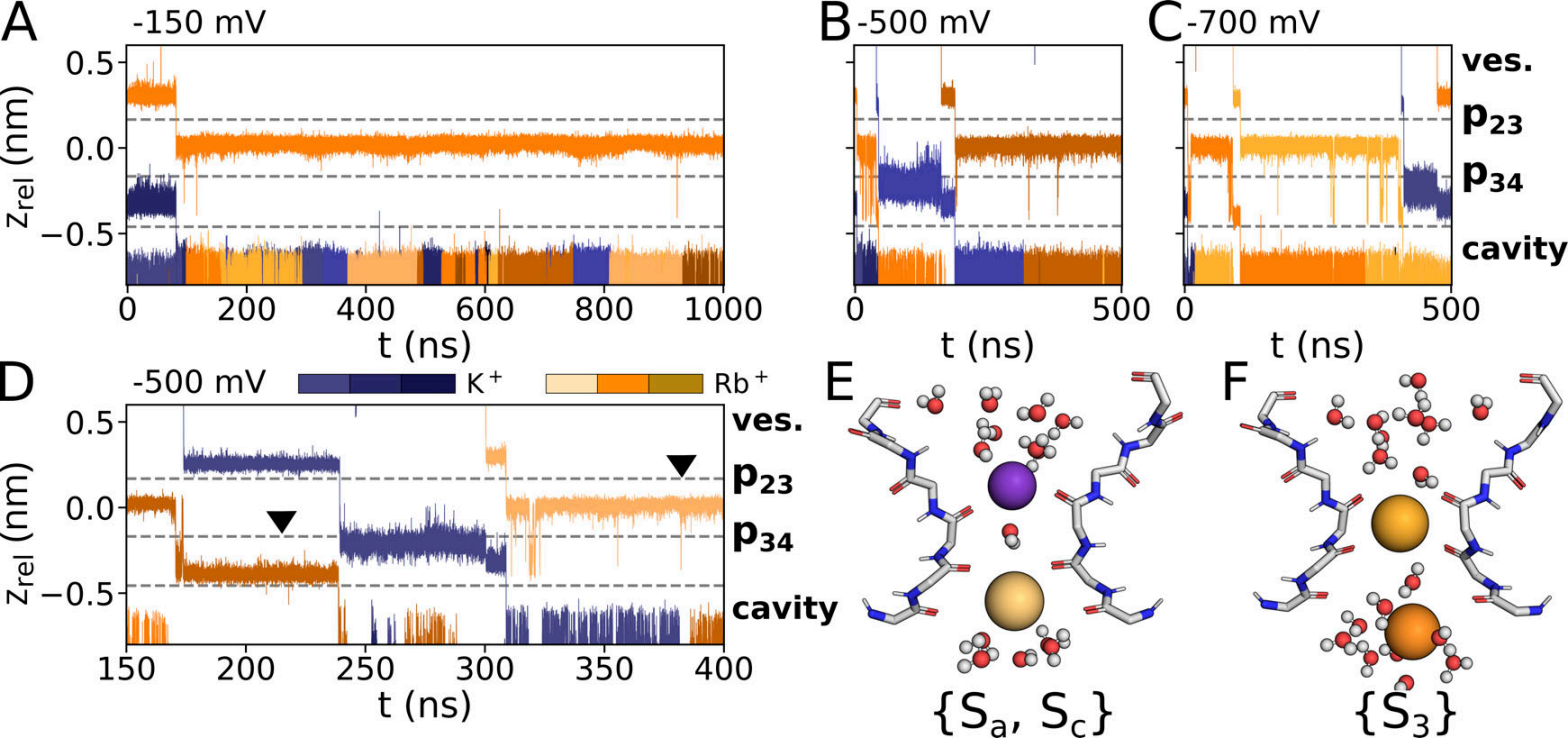
**Preferred binding sites of Cs**^**+**^ **within the SF. (A–C)** Conduction trajectory for Cs^+^ along SF axis from simulations in mixed-ionic solutions with K^+^, at different voltages and for different initial cation placements within the SF: Cs^+^ is preplaced in p23 and K^+^ in p34 at simulations with an applied voltage of (A) −150 mV, (B) −500 mV, and (C) −700 mV. **(D–F)** The conduction pattern of Cs^+^ and K^+^ is depicted in D for a simulation at −500 mV, with the initial placement of K^+^ proximate to p23 and of Cs^+^ proximate to p34. Snapshots of trajectory frames at timepoints indicated by arrowhead markers in D are shown in subplots (E and F).

Further simulations were performed in 900 mM CsCl at −700 mV. A typical trajectory is shown in Fig. 2 C, in which three ions are fully permeating the filter in a 500-ns long simulation. The number of Cs^+^ transitions is much lower than the conduction events of K^+^ or Rb^+^ under the same condition (e.g., eight permeations for Rb^+^ at −700 mV; Fig. 2 B). Fewer permeations were also observed for lower voltages, with no detectable permeation at −150 mV in a simulation run over 1 µs (Fig. 5 A).

The data show that the filter can again contain either a single cation or two cations at the same time, both for pure Cs^+^ and mixed K^+^/Cs^+^ solutions (Fig. 2 C; and Fig. 5, B and D). In the two-ion state, the scenario is similar to the K^+^ solution with cations in a {Sa, Sc} state (Fig. 2 C). After the lower ion leaves the cavity, the upper ion moves from Sa into S3. While K^+^ remains only shortly in the S3 position, Cs^+^ stays there for long periods of time. The occupation of S3 is very stable at moderate negative voltages and at voltages ≤−500 mV; only interrupted by occasional short excursions into Sc (Fig. 5, B–D; Fig. S4; and Fig. S5). At these high voltages, the Cs^+^ is only expelled from S3 when a new ion—either Cs^+^ or K^+^—enters the filter from the bulk solution. The Cs^+^ ion in the filter can then move into the cavity by crossing p34 in Sb. The different modes of filter permeation by K^+^ and Cs^+^ are clearly seen in simulations with mixed Cs^+^/K^+^ solutions and a voltage of −500 mV (Fig. 5 D). While K^+^ moves in sequential steps from Sa via Sb to Sc below p34, Cs^+^ exhibits a long-lasting intermediate step at S3. Moreover, unlike K^+^ and Rb^+^, Cs^+^ occupied S3 in prolonged single-cation states at all voltages, whereas binding to site Sb was not observed at all (Fig. 5, B–D and F). Like for Na^+^, K^+^, and Rb^+^, the {Sa, Sc} state was observed for both Cs^+^–K^+^ as well as Cs^+^–Cs^+^ exchanges (Fig. 5 E). {Sa, Sc} states were short-lived compared with {S3} states at the same voltage, with >900 ns at −150 mV, indicating {S3} as an energetically favorable ion configuration of Cs^+^ in the SF.

**Figure 6.**
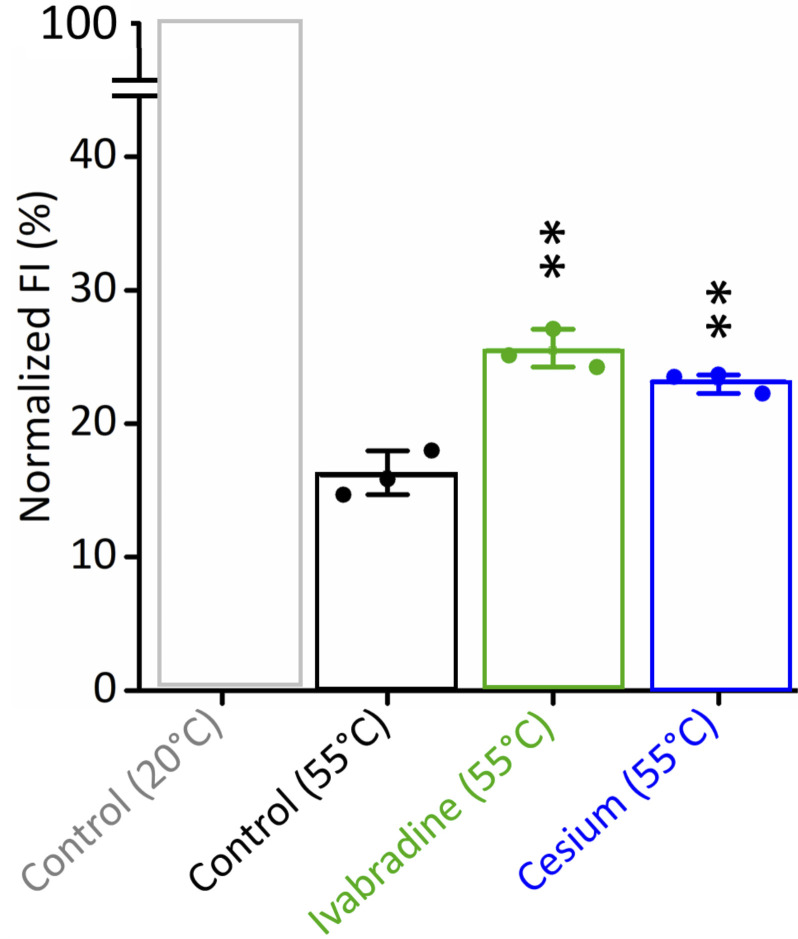
**HCN4 is equally stabilized by the open pore blocker Ivabradine and by Cesium.** Relative fluorescence intensity (FI) for GFP-HCN4 protein samples heated for 10 min at 20°C (control: gray, 100%) and the same protein heated at 55°C in the absence (black, 16.2 ± 1%) or presence of 0.5 mM Ivabradine (green, 25.5 ± 0.8%) or 50 mM CsCl (blue, 23.1 ± 0.4%). Normalized FI values are mean of *n* ¼3 SEM. Statistical analysis performed with one-way ANOVA, followed by Fisher’s test (**P < 0.001).

Because of the unexpected conductance for Cs^+^ in simulations at −500/−700 mV, we performed control experiments for testing the relevance of the cryoEM structure for Cs^+^ conductance/block. We also critically evaluated the quality of our computational model in general and for Cs^+^ transport in particular. A first assay addressed the question if the structure of the open HCN4 pore (Saponaro et al., 2021a), which is the basis of the present simulations, is reflecting the voltage-activated or the constitutively open state (Accili, 2022). The finding that Cs^+^ only blocks in electrophysiological recordings the former but not the latter open state (Proenza et al., 2002) predicts that Cs^+^ ions should only be bound in the selectivity filter if the protein is in the activated open state. We tested Cs^+^ binding in the filter by a thermal stability assay assuming (Saponaro et al., 2021b) that the structural integrity of the protein during thermal denaturation should only be stabilized when Cs^+^ is effectively bound in the filter. The HCN4 protein was therefore purified under the same conditions that were used for obtaining the open pore structure (Saponaro et al., 2021a). The data in Fig. 6 show that the presence of the known HCN pore blocker Ivabradine, which was used as the positive control, causes the expected protection of the channel protein against melting (Saponaro et al., 2021b). An equivalent stabilization of the protein is obtained by Cs^+^ suggesting that also this cation is binding (presumably inside the pore) and stabilizes the protein. The results of these experiments provide indirect evidence that the protein isolation procedure shifts the HCN4 pore into a state, which resembles the voltage-activated and Cs^+^-blocked open state (Saponaro et al., 2021a). This is in agreement with computational results in which the cation binds inside the selectivity filter and exhibits long residence times in S3.

**Figure 7.**
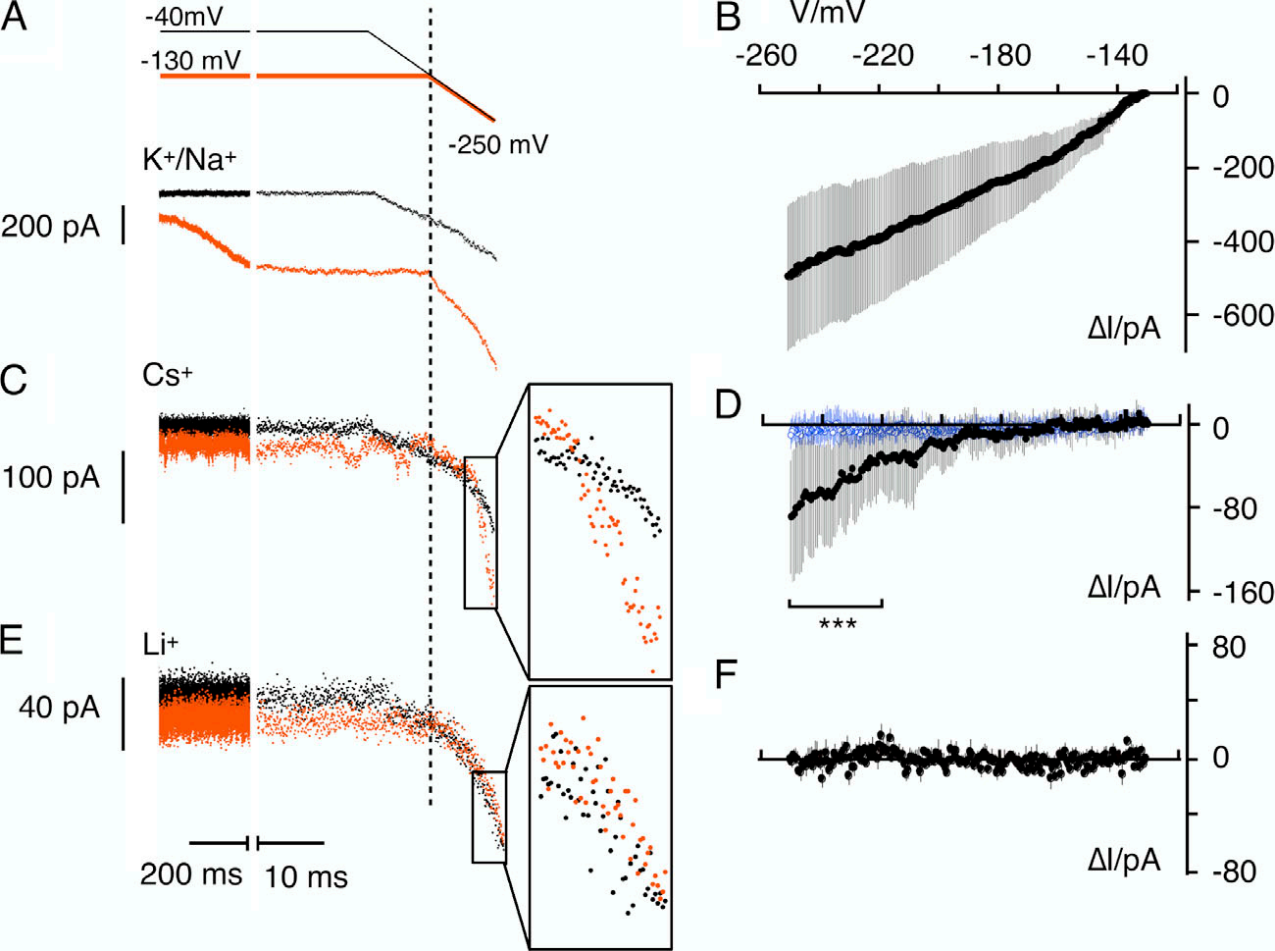
**Cation conductance of HCN channels at extreme negative voltages.** Membrane currents of HEK293 cells constitutively expressing HCN2 were measured in whole-cell configuration in buffers with different cations. Cells were clamped to a two-stage voltage protocol (top panel) comprising of 712- or 720-ms-long voltage steps from resting voltage to either −40 mV (black) or −130 mV (orange), respectively, followed by a fast ramp (12 mV/ms) from −40 or −130 to −250 mV. **(A, C, and E)** Membrane currents elicited in individual HCN2 expressing HEK293 cells by voltage protocol with prepulse to −40 mV (black) and −130 mV (orange) in standard buffer (110 mM NaCl/30 mM KCl; A), 140 mM CsCl (C), and 140 mM LiCl (E). Boxed currents in C and E are magnified in insets. **(B, D, and F)** Mean ∆I_HCN_/V relationships in standard buffer (B), 140 mM CsCl (D), and 140 mM LiCl (F). ∆I/V data were obtained by subtracting ramp currents following prepulse to −40 mV from respective currents after prepulse to −130 mV after normalizing to currents at −130 mV. Black data points are means ± SD from HNC2 expressing HEK293 cells in standard medium (*n* = 10), 140 mM CsCl (*n* = 15), and 140 mM LiCl (*n* = 5). Blue data points are mean I/V relations (*n* = 7) obtained with same procedure as in C and D, but in control HEK293 cells not expressing HCN channels. Currents in D between −220 and −250 mV are significantly different (P < 0.001) between HCN2 expressing cells and non-expressing control cells.

Finally, we tested if the computationally predicted permeation of Cs^+^ at high negative voltages can be experimentally confirmed. It is known that high voltages can force—by a so-called punch-through mechanism—the transition of otherwise impermeant ions through the SF of K^+^ channels (Mita et al., 2021; Nimigean and Miller, 2002). To examine the possibility of a Cs^+^ punch through at high negative voltages in HCN channels, we measured the conductance of HCN2 in HEK293 cells in extracellular solution with 110/30 mM NaCl/KCl or 140 mM CsCl. Currents in HEK293 cells stably expressing HCN2 were recorded in response to a voltage step from the resting voltage to either −40 or −130 mV followed by a fast voltage ramp to −250 mV. Fig. 7 A shows a typical example of a cell in a standard buffer where the prepulse to −40 mV elicits the small endogenous background currents and the current through constitutively open HCN channels (black). The prepulse to −130 mV evokes in addition to the latter the slow voltage-activated component of the HCN channel (orange). Currents elicited by the fast ramp following the preconditioning to −40 mV should reflect the open channel current of endogenous channels and constitutively open HCN channels (I_pc-40_). The latter plus the open HCN channels activated by voltage should dominate the ramp current (I_pc-130_) following a prepulse to −130 mV (Fig. 7 A). To obtain the current/voltage relation of the voltage-activated HCN channels (I_HCN_/V), I_pc-40_ was subtracted from I_pc-130_ after normalizing the ramp currents to the current at −130 mV. The mean ∆I_HCN_/V relation (±SD, *n* = 10 cells) measured in the standard medium is plotted in Fig. 7 B. The ∆I_HCN_/V relation of the open HCN2 channels, which were activated at −130 mV is as expected approximately linear; the small tendency of saturation presumably reflects saturation of the unitary channel conductance at extreme voltages.

The procedure was repeated in a 140-mM CsCl buffer showing typically little current in response to both prepulse to −40 and −130 mV (Fig. 7 C). This reflects the fact that Cs^+^ is not conducted by HCN channels over this voltage window even though the channels are presumably activated at −130 mV. A comparison of the currents elicited by the voltage ramps shows that I_pc-40_ and I_pc-130_ are indistinguishable at voltages between −130 mV and approximately −200 mV. At more negative voltage, I_pc-130_ progressively exceeds I_pc-40_ (Fig. 7 D). The same behavior was confirmed in 14 additional cells generating an inward rectifying mean ∆I_HCN_/V relationship (Fig. 7 D). We interpret this inward current as evidence for a small Cs^+^ inward conductance at voltages more negative than −200 mV through the voltage-activated portion of the HCN channels. The current amplitude of the Cs^+^ inward current at −250 mV is approximately five times smaller than the current measured in the standard Na^+^/K^+^ buffer, suggesting that the Cs^+^ current generated by punch through is much smaller than that of the conducted K^+^ and Na^+^.

The assumption of a Cs^+^ punch through current in HCN channels at high voltages in a buffer with a high Cs^+^ concentration was confirmed by experiments in which the same procedure was performed in seven untransfected HEK293 cells. In this case, the ∆I/V relation is close to zero over the entire voltage window (Fig. 7 D, blue symbols), suggesting no voltage-dependent inward current in the absence of HCN channels; the currents negative of −220 mV are in the control cells significantly smaller than those in the HCN2-expressing cells.

Since the MD simulations predict that Li^+^ is also not transported by HCN channels (Figs. 2 and 3), the same experiments as in Fig. 7, A–D, were repeated in five HCN2-expressing HEK293 cells with 140 mM Li^+^ in the external buffer. In these experiments there is, like in the control cells, no apparent difference between I_pc-40_ and I_pc-130_ (Fig. 7, E and F). The ∆I_HCN_/V relation confirms that the voltage-activated portion of HCN2 is under these conditions not conducting Li^+^—even at extreme negative voltages and again in good agreement with our computational data.

### Probability density profiles and residual times in the filter indicate S3 as a binding site for Cs^+^ inhibition

**Figure 8.**
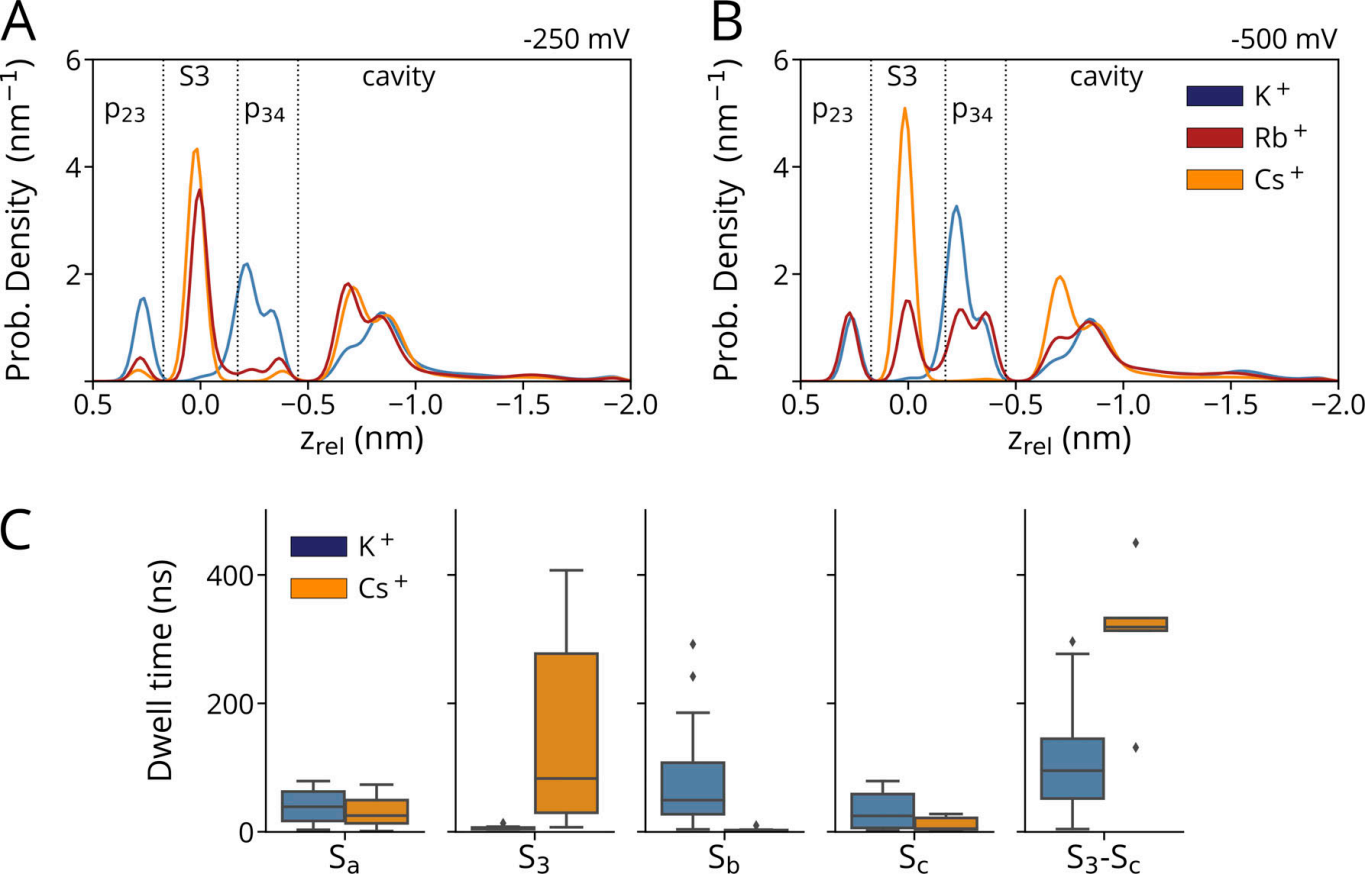
**Different cations occupy filter binding sites with different propensity. (A and B)** Probability density profiles for K^+^ (dark blue), Rb^+^ (red), and Cs^+^ (orange) in SF at −250 mV (A) and −500 mV (B). The density profiles were computed as kernel density estimates for aggregated simulations at both voltages. **(C)** Boxplots depicting the dwell time of K^+^/Cs^+^ in SF sites at −500 mV. Boxes span the interquartile range between Q1 and Q3, whiskers denote the 1.5 interquartile distance, while outliers are shown as single points. SF positions were assigned using filtered ion trajectories (see Materials and methods). Data shown in A and B were obtained from simulations conducted with pure solvated KCl (*n* = 3 at −250 mV, *n* = 4 at −500 mV), RbCl (*n* = 3 at −250/−500 mV), and CsCl (*n* = 2 at 250 mV, *n* = 3 at −500 mV), with a simulation time of 500 ns each. Results for C were obtained from simulations with KCl and mixed KCl/CsCl at −500 mV (*n* = 4 for both).

The different translocation mechanisms of the three cations are best illustrated by an analysis of their probability density profiles in individual filter binding sites along the main axis of the channel pore. The most pronounced difference is observed between K^+^ and Cs^+^: the smaller K^+^ ion is rarely seen in S3 at voltages of −250/−500 mV, but frequently in p23 and p34. The larger Cs^+^ ion exhibits the inverse behavior: at both voltages, it is mostly occupying S3, but not the carbonyl level in Sa and Sb. The intermediate size Rb^+^ ion is a hybrid between K^+^ and Cs^+^. At the lower voltage it resembles the binding pattern of Cs^+^, while at higher voltages it tends to mimic the behavior of K^+^ (Fig. 8, A and B).

The data suggest a scenario in which Cs^+^ can permeate the HCN4 selectivity filter after binding to Sa, where it finds in S3 its preferred binding site. Both a tight binding to S3 as well as an energetically unfavorable transition through p34 in Sb could be the rate-limiting step in the translocation of Cs^+^, which is effectively blocking the permeation of K^+^ through the SF. To further examine this hypothesis, we estimated the absolute residence time distributions of Cs^+^ and K^+^ in different filter binding sites. The data from ion trajectories along the z-axis were filtered using the Butterworth method to reduce fast fluctuations prior to entering a binding site. Since Cs^+^ and K^+^ are not binding to the same extent to the same sites, we estimated the pooled residence time of both ions in the combined set of identified binding sites (Sa, S3, Sb, and Sc). The main information from the data in Fig. 8 C is that the median residence time of Cs^+^ ions in the narrow part of the filter (Sa–Sc) is approximately six times higher than that of K^+^.

Collectively, the data suggest an increasing preference for cations to bind to S3 and Sc instead of Sb with increasing ionic radius. Since Rb^+^ and Cs^+^ show a cation-specific propensity to remain bound to S3, and since this phenomenon is voltage-dependent, it is reasonable to speculate that this mechanism is underlying the differential blocking efficiency of both cations. From experiments, it is known that Cs^+^ exhibits a strong and Rb^+^ a weak voltage-dependent block of native I_f_ currents at negative voltages (DiFrancesco, 1982).

### Cation-induced fit of filter geometry

It has been reported that the selectivity filter of HCN channels is less rigid than the corresponding domain of canonical K^+^ channels (Ahrari et al., 2022; Saponaro et al., 2021a). The former can apparently adapt to the type of cations that are entering the SF. As a result of such an ion-induced widening of the p34 site, a K^+^ ion can acquire the position in the plane between the carbonyl oxygens in Sb, a position only accessible to the smaller Na^+^ ion in selective K^+^ channels. A current view is that this interplay between ions and protein is the basis for the weak K^+^/Na^+^ cation selectivity of HCN channels in that a K^+^-induced widening of the filter releases Na^+^ from a strong binding site in Sb and promotes the permeation of K^+^ and Na^+^ in this manner (Bauer et al., 2022).

**Figure 9.**
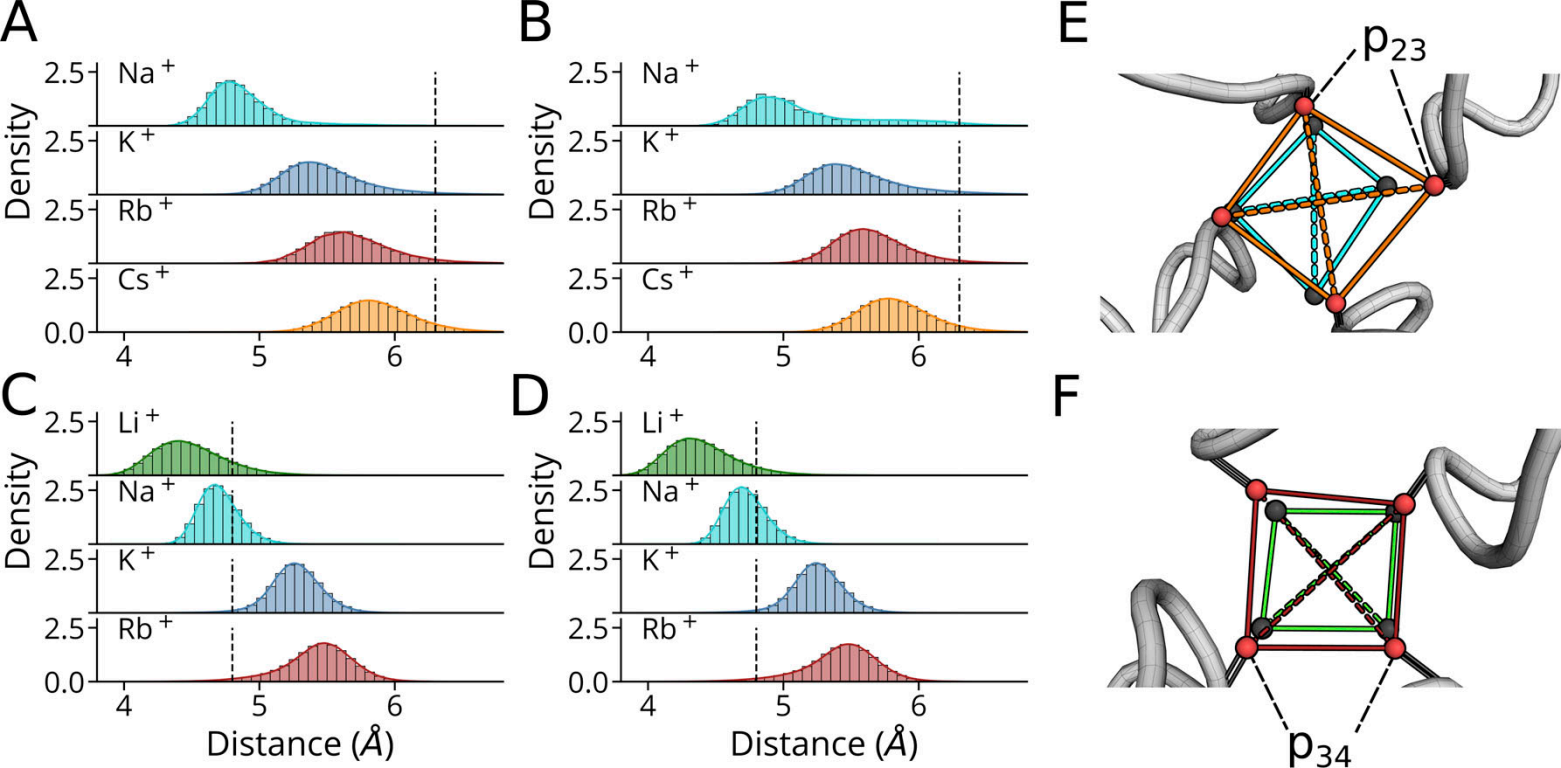
**Cation-dependent modulation of distances between filter carbonyl oxygens. (A–D)** Inter carbonyl-oxygen distance histograms and overlayed KDE plots for different cation species in p23 are depicted for (A) subunits A|C and (B) B|D, distances between these subunits in p34 are shown in C and D. Reference distances derived from the cryoEM structure are depicted as black dashed lines. Distances were extracted from simulations conducted between −150 and −700 mV for the HCN4 pore domain. Only distances associated with binding events lasting ≥12 ns are considered. Counts in histogram bins were normalized to yield a total histogram area equal to 1. **(E and F)** Representative frames for the largest and smallest cations bound and associated carbonyl oxygen rearrangements are shown for (E) p23 with Na^+^ and Cs^+^ and (F) p34 with Li^+^ and Rb^+^. Here, carbonyl groups in p23/p34 are shown as stick representation, with red spheres at the position of carbonyl oxygens in the widened state (i.e., with Cs^+^ and Rb^+^ proximate to p23 and p34, respectively). Gray spheres indicate the carbonyl oxygen position for states with Na^+^ and Li^+^ in p23 and p34, respectively. Carbonyl oxygen planes are indicated by solid lines, and ∆p23 and ∆p23 values for both subunits are indicated by dashed lines with cyan for Na^+^, green for Li^+^, red for Rb^+^, and orange for Cs^+^.

**Figure 10.**
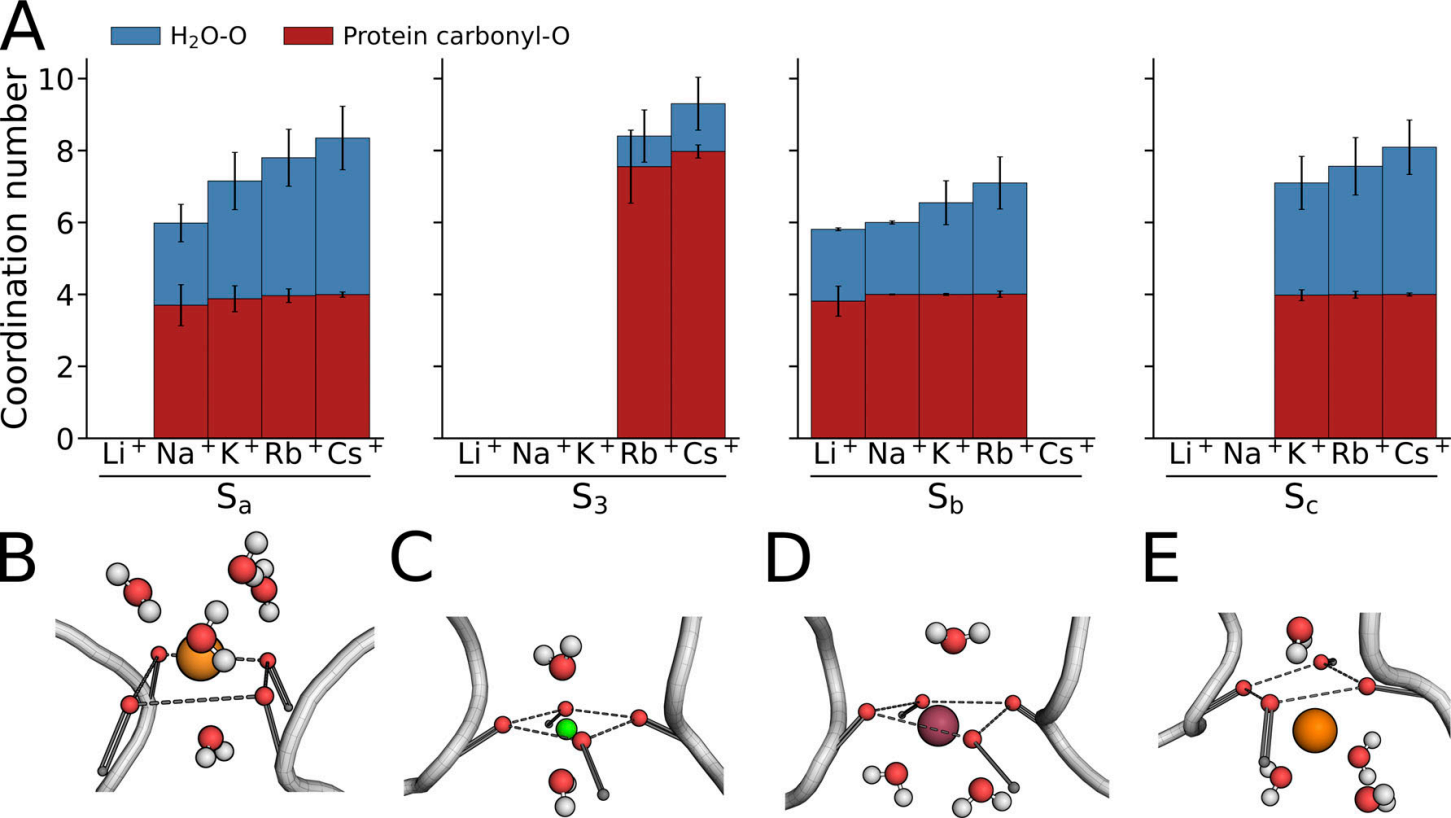
**Different cations exhibit distinct numbers of coordination oxygens from amino acid carbonyls and water. (A)** Time-averaged number of coordinating oxygen atoms for sampled cation species in different SF binding sites drawn from MD simulations conducted at voltages between −700 and −250 mV in pure-cationic solution (900 mM): −700 mV (*n* = 4) for Li^+^, −500 mV (*n* = 2) for Na^+^, −250 mV (*n* = 3), and −500 mV (*n* = 4) for K^+^, −250 mV (*n* = 3), −500 mV (*n* = 4), and −700 mV (*n* = 1) for Rb^+^, −150 mV (*n* = 2), −250 mV (*n* = 2), −500 mV (*n* = 3), and −700 mV (*n* = 2) for Cs^+^. For further sampling with Li^+^ and Na^+^, a subset of mixed-cationic simulations was also included: −700 mV (*n* = 1) for Li^+^/Na^+^, −700 mV (*n* = 3) for Li^+^/Na^+^/K^+^, and −500 as well as −700 mV (*n* = 1 each) with Na^+^/K^+^. Error bars indicate the standard deviation. **(B–E)** Simultaneous binding by carbonyl oxygens and H_2_O oxygens in Sc is depicted by representative trajectory frames for Cs^+^ in Sa (B), Li^+^ (C), and Rb^+^ I (D), as well as Cs^+^ in Sb (E). Two opposite monomers are shown in the tube representation, carbonyl oxygens involved in cation binding are depicted as red spheres. Dashed gray lines indicate the oxygen plane.

To further examine this structural adaptation of the filter in HCN4 to different cation species, we measured the interchain distances between carbonyl oxygens in opposing monomers (A|C; B|D) in p23 and p34 (Δp23/Δp34) for binding events lasting ≥12 ns in simulations conducted at hyperpolarizing voltages. The resulting histograms are shown in Fig. 10, A–D. Species with spurious dwell time proximate to carbonyl oxygen planes, namely Li^+^ in p23 within Sa and Cs^+^ in p34 within Sb, were not considered for that specific site. The measured carbonyl oxygens distances in Fig. 9 show the general tendency of the filter to dynamically adapt with both binding sites to all different bound cation species. In both subunit pairs Δp23 and Δp34 increase as a function of the increasing ionic radius of the cations (Fig. 9, E and F).

In an additional step, we also compared the same distances induced by different cations with the respective distance from the cryoEM structure of HCN4; the latter was obtained with a Na^+^ ion in the SF (Saponaro et al., 2021a). This analysis shows that the binding of alkali metal cations with a higher periodic number than Na^+^ causes an increase in Δp34. Binding of the smaller Li^+^ on the other hand decreases the Δp34 value. In this respect, the pattern of cation/filter interactions in p34 differs from the p23 site. In the latter, all cation species reduced the Δp23 value with respect to distance in the cryoEM structure; in this case, the largest decrease in Δp23 occurs with the smallest bound cation, Na^+^.

In the context of HCN function, the present data bear interesting information on the functional features of the channel. The distance values as well as the short dwell times for Cs^+^ in Sb and Li^+^ in Sa suggest a limited degree of structural adaptation of the p34 and p23 sites to accommodate cations larger than Rb^+^ and smaller than Na^+^, respectively. Hence, p23 effectively acts as an SF-entrance barrier for Li^+^. In the same line of thinking, Cs^+^ presumably remains for long times in S3 because p34 is not able to accommodate this cation unless the voltage becomes high enough to force the ion through. This in turn leads to an attenuation or blockade of K^+^ conduction.

The data shown in Fig. 9 suggest a simple relationship between cation size and selective binding to the SF. However, it is important to mention that also the hydration number of alkali metal cations increases with the period number (Table S6) and has been frequently discussed in the literature as a crucial factor that determines the cation selectivity of the filter in ion channels (Thomas et al., 2007; Varma and Rempe, 2007). To examine the cation interactions in the HCN4 filter, the time-averaged number of coordinating H_2_O or p23/p34 backbone carbonyl oxygens in filter subregions was determined from MD simulations with single cation species (Fig. 10 A). Overall, the total number of coordinating atoms increased from an SF site-aggregated median value of 6 for Li^+^ (mean: 5.8) and Na^+^ (6.0), over 7 for K^+^ (6.8), 8 for Rb^+^ (7.9), and 9 Cs^+^ (9.0). A nearly equal contribution of H_2_O oxygens and backbone carbonyl oxygens was observed in sites Sa, Sb, and Sc, whereas cations were bound to a high number of coordinating backbone oxygens in S3. This emphasizes the role of additional coordinating H_2_O oxygens for binding to the oxygen planes and the wide Sc site in HCN4.

It is interesting to note that the number of coordinating H_2_O molecules is higher in site Sa compared with Sb. The higher coordination number for Rb^+^/Cs^+^ in the Sa-bound state is presumably the consequence of an asymmetry of the pore in which the vestibule volume is much wider than the cavity; this favors an H_2_O accessibility of Sa compared with Sb. In S3, which exhibits no prolonged binding of the smaller Li^+^, Na^+^, and K^+^, eight carbonyl oxygens participate in the binding of the larger Rb^+^ and Cs^+^. In contrast, p34 in the Sb site, which excludes Cs^+^ binding, seemingly accommodates cations with a maximal hydration number of approximately seven. In Sa and Sc, additional binding sites for Cs^+^ are provided by H_2_O molecules. Overall, these results seem to indicate a bias of cations with higher coordination numbers for binding Sa/S3/Sc over Sb. Also, comparisons between Li^+^ and Na^+^ provide additional insights: Na^+^ is bound to both Sa and Sb with a mean coordination number of approximately six, while Li^+^ binds Sb with the same coordination number but is unable to bind Sa. This implies that the Sa site and the surrounding water molecules are unable to stably provide because of geometric demands of the protein in p23/Sa (Fig. 9, A and B), the required six coordination sites for Li^+^.

Collectively, our results suggest that the binding of cations to different sites in the HCN4 filter induces distinct filter deformations which in turn induce favorable binding positions. In such an induced-fit type scenario, the cation species are coordinated by a cation-specific number of oxygens, either donated by the protein backbone or H_2_O molecules. This kind of interplay, which is governed by the number of available coordinating oxygens at a given filter binding site and the resulting mechanical strain on the filter residues for accommodating a cation in the SF is not unique to the HCN filter. It was proposed that the same physicochemical interactions between the ion and protein are determining the interaction energy and thus the selectivity of filters for different cations in ion channels with flexible filters (Yu et al., 2010). The main difference between HCN and canonical K^+^ channels in this context is the higher flexibility of HCN4 filter residues and greater accessibility for H_2_O molecules to the binding sites. Both the negligible conductance for Li^+^ and the blockade of Cs^+^ are likely caused by a sterically limited adaptation of carbonyl oxygens in binding sites Sa and Sb, respectively, resulting—together with proximate H_2_O molecules—in a non-optimal number of coordinating oxygen atoms for both species.

## Discussion

The conducting and blocking features of metal cations in HCN channels are well-known from electrophysiological recordings of I_f_/I_h_ and HCN currents, and these properties are recapitulated in the present MD simulations with the open HCN4 pore. This includes the fact that Li^+^ is neither appreciably conducted by HCN channels nor inhibiting K^+^ currents of these channels (D’Avanzo et al., 2009; Ho et al., 1994; Wollmuth and Hille, 1992). Also, the experimentally known Rb^+^ conductance of HCN channels, which is in a similar order of magnitude as the K^+^ conductance (D’Avanzo et al., 2009; Wollmuth and Hille, 1992), is evident in the simulations. The unexpected inward transitions of Cs^+^ ions in simulations at high negative voltages were at first glance not expected from the experimental findings, in which Cs^+^ shows a voltage-dependent block of I_f_ currents and HCN channels (DiFrancesco, 1982; Moroni et al., 2000). But our experimental data confirm the prediction from the simulations in that high voltages cause a punch through allowing some Cs^+^ conductance at extreme voltages. This favorable agreement between experimental data and simulation results underpins that the open pore structure of HCN4 is a suitable model for studying structure/function correlates in these channels. The fact that the attenuation of K^+^ current by Cs^+^ is a property of the voltage-activated open state but not of the constitutively open state of the channel (D’Avanzo et al., 2009; Proenza et al., 2002) suggests that also the cryoEM structure of the HCN4 channel reflects the channel in this activated open state and not in a constitutively open state since the latter is insensitive to Cs^+^ block.

With this good agreement between computational and experimental data, we can now extract information on the mechanisms of ion discrimination by the SF of these channels. The lack of Li^+^ inward conductance and the absence of a Li^+^ block can be well explained by the fact that Li^+^ is unable to spontaneously enter even the outermost binding site (p23) from the external medium at hyperpolarizing voltages. In this way, Li^+^ has no negative impact on the conductance of K^+^ and Na^+^ ions in the simulations as well as in physiological measurements because it is not able to reach from the external solution the binding site p34 in which it can stably bind. Hence, a differential binding of Na^+^ and Li^+^ to p23 is the molecular determinant for the difference in selectivity between the two cations in HCN channels.

The simulations show that the HCN4 pore conducts in a pure Rb^+^ solution and in mixed Rb^+^/K^+^ buffers Rb^+^ to a similar degree as K^+^. But the larger Rb^+^ occupies in a voltage-dependent manner different binding sites from K^+^ in the filter. As a unique feature, Rb^+^ binds at lower voltages like the larger Cs^+^ to S3. But with increasing negative voltage, Rb^+^ then rapidly fluctuates between binding to S3 and p34, a behavior not seen for any of the other cations. The data are not providing a mechanistic explanation for the small inhibitory effect of Rb^+^ on the K^+^ current. But the fact that Rb^+^ is conducted in the filter in a different manner from K^+^ is already a first indication for a potential slowdown of K^+^ conductance by Rb^+^. The fact that the occupation of Rb^+^ in the filter is voltage-dependent implies that this behavior could be the basis for the shallow voltage-dependent block of K^+^ currents (DiFrancesco, 1982).

It is well established that I_f_/I_h_ currents as well as HCN channels are blocked in a voltage-dependent manner by sub-millimolar concentrations of Cs^+^ in the external medium (DiFrancesco, 1982; Moroni et al., 2000). The most important observation in this context is that Cs^+^ shows a peculiar transition pattern in that it remains for a very long time in the S3 site. Because of this long residence in S3, Cs^+^ reduces the frequency of K^+^ transitions in mixed solutions. This scenario can be interpreted as the expected mechanisms of Cs^+^ block of HCN channels. The simulation data further indicate that binding of Cs^+^ in S3 can be overcome by very negative voltages, suggesting a punch trough in which a voltage-dependent block is partially released at very high voltages. The fact that this prediction could be confirmed in experiments with HCN2 channel underpins the correctness of the model. In relation to Cs^+^ asymmetrical effects discussed above, it is worth noting that experiments on native funny channels in Purkinje fibers, while showing a strongly voltage-dependent block at negative voltages, revealed a still-unexplained increase of I_f_ at positive voltages upon external addition of millimolar concentrations of Cs^+^ (DiFrancesco 1982). A similar, more marked behavior was found with Rb^+^. The results reported here revealing a cation-dependent modification of filter geometry may provide a potential explanation if we assume for example that occupation of S3 by Cs^+^ or Rb^+^ facilitates K^+^ transition from S4 to the extracellular side.

In patch-clamp experiments, it was predicted on the basis of the Woodhull model that the electrical distance between the blocking site for Cs^+^ and the outer membrane surface relative to the membrane thickness is independent of the K^+^ concentration and has a value of about 0.7 (DiFrancesco, 1982; Moroni et al., 2000; Woodhull, 1973). Both experimental findings are in good agreement with the view of the S3 site as the main Cs^+^ blocking site. The electrical field across K^+^ channels does not drop over the entire membrane but mostly across the narrow part of the filter (Kopec et al., 2019). With this information, we estimate from Fig. 5 F an equivalent value for the relative distance of about 0.6. The latter is obtained from the position of the S3 site relative to the length of the narrow filter. Both values are sufficiently similar considering that we neither know the precise length in the structure over which the electrical field drops nor whether this drop is linear. But as predicted from the physiological measurements, this value is not affected by K^+^; in the simulations, Cs^+^ binds to S3 in the presence and absence of K^+^ and the position of S3 relative to the length of the filter remains in both conditions unaffected.

Selectivity filters of HCN channels have the peculiar feature of being more flexible than the rigid filters of canonical K^+^ channels; the narrow filter geometry of the critical inter-carbonyl-oxygens can in the HCN4 and HCN1 pores open up such that even a K^+^ ion can enter sites which are in canonical K^+^ channels reserved for the smaller Na^+^ (Ahrari et al., 2022; Saponaro et al., 2021a). This K^+^-induced widening of the filter presumably also widens the distance of critical inter-carbonyl-oxygens with the effect that Na^+^ ions, which are kept in the respective sites, are released for further transport. This general picture of cation-selective filter dynamics in the filter of the HCN channel is confirmed by the response of the narrowest binding sites p23 and p34 to all cations tested. The data show that the distances between the carbonyl oxygens, which participate in the binding sites p23 and p34, increase as a function of the periodic number of the cations binding these sites. In search for a physico-chemical feature, which drives this distinct binding of cations and the resulting size adaptation of the binding sites, we find that each cation exhibits in its binding sites always a characteristic number of coordinating oxygens; the latter increases with the periodic number and the size of the cations. This oxygen coordination is provided in different binding sites to different degrees by an interaction of the cation with water or carbonyl oxygens from the protein. In the S3 site, the major contribution comes from interactions with the protein, while in the other sites, water and protein interactions are equally important. It had already been proposed from work on canonical K^+^ channels that cation selectivity is determined by a combination of the capability of the filter residues to model energetically favorable H_2_O coordination patterns around solvated cations and the mechanical strain associated with these rearrangements on filter residues (Mita et al., 2021; Noskov and Roux, 2007; Thompson et al., 2009; Yu et al., 2010).

The rigid filter architecture of canonical K^+^ channels apparently limits the formative impact of different cations on the local structure of the binding sites. Consequently, Cs^+^ and Rb^+^ ions occupy in the crystal structures of KcsA the same sites as K^+^ (Montoya et al., 2017). Because of the unique flexibility of the HCN filter, the selectivity of the latter channels is determined by mutual filter/cation interactions. In this scenario, the propensity of cations to bind to S3 increases in a sequential manner with the increasing coordination number of the cations. Consequently, the propensity of Sb-binding and finally conduction decreases. It is tempting to speculate that the p34 plane within Sb is not providing a suitable oxygen coordination environment together with neighboring H_2_O oxygens in S3 and Sc for Cs^+^. The latter therefore remains in S3 and blocks in this way the Na^+^/K^+^ inward current.

### Conclusion

Taken together, our data suggest that the cation selectivity of the HCN4 filter is determined by the differential binding of different cations to three main SF substructures, namely p23, S3, and p34. These cation-specific binding patterns are the basis for both the discrimination between Li^+^ and Na^+^ conductance and for the channel block by Cs^+^. We observe a simple trend according to which different cation species prefer binding to S3 over p34 with an increasing period number. The filter residues, therefore, rearrange together with solvent water molecules to create a characteristic local coordination environment that is suitable for each cation species: When bound to a given site, the cations are coordinated by a distinct number of oxygens, which is characteristic for each cation species. The required oxygens are either donated by filter residues or H_2_O molecules in the vicinity of the filter. The geometric adaptations of filter residues to the binding of different cation species seem to be limited by the capability of filter residues to accommodate a given cation in p23/p34. This is indicated by the cation-specific deviations of the filter geometry in the MD simulations from the cryoEM structure. It appears as if both factors, namely the available coordination sites at a given SF substructure and the mechanical strain induced by the cation, are mutually interacting for generating the HCN4 typical conduction pattern. In this context, the fact that HCN4 conducts Na^+^ but not Li^+^ can be explained by the ability of p23 to bind Na^+^ but not Li^+^. Along the same line of thinking the prolonged binding of Cs^+^ to S3 and the consequent attenuation of K^+^ currents is most likely caused by an energetically unfavorable Cs^+^ transition through p34. This energy barrier can be overcome by extreme negative voltages causing a punch-through in the simulations as well as in experiments. Effectively, the HCN4 SF geometry limits conductance to cations that are capable of binding to p23/p34 and can traverse through S3. This is exemplified by Rb^+^ with a voltage-dependent binding pattern resembling either K^+^ or Cs^+^.

## Supplementary Material

Table S1 shows an overview of equilibration steps prior to production MD runs with protein backbone and sidechain restraints for successive NPT steps.

Table S2 shows the summary of simulations with pure cationic solutions.

Table S3 shows a summary of simulations with mixed-cationic solutions containing Li^+^.

Table S4 shows summary of simulations with mixed-cationic solutions containing Rb^+^ or Cs^+^.

Table S5 sshows shows the summary of simulations drawn from Saponaro et al. (2021a) and Bauer (2021).

Table S6 shows literature values for the size and hydration number of tested monovalent cations.

## Data Availability

Input files for MD simulations as well as data underlying graphs depicted in Figs. 2, 3, 4, 5, 8, 9, and 10 are available together with exemplary scripts for data visualization via Zenodo (https://doi.org/10.5281/zenodo.8122465). MD trajectories and additional scripts for data analysis are available from K. Hamacher (hamacher@bio.tu-darmstadt.de) upon reasonable request.
