# Supplementary material for: Alkali metal cations modulate the geometry of different binding sites in HCN4 selectivity filter for permeation or block
Source: J Gen Physiol. 2023 Jul 31;155(10):e202313364. doi: 10.1085/jgp.202313364 (PMC10386491; doi:10.1085/jgp.202313364)
Supplement: Table S1 — shows an overview of equilibration steps prior to production MD runs with protein backbone and sidechain restraints for successive NPT steps. [file JGP_202313364_TableS1.docx]

**Equilibration procedure – Table**

| **Ensemble** | **NVT** | **NPT** | | | | |
| --- | --- | --- | --- | --- | --- | --- |
| **t_sim_ (ns)** | **0.1** | **20** | **1** | **1** | **1** | **1** |
| **BB restraints (kJ mol^-1^ nm^-2^)** | **1000** | **1000** | **1000** | **500** | **200** | **50** |
| **SC restraints (kJ mol^-1^ nm^-2^)** | **1000** | **1000** | **500** | **200** | **50** | **0** |

***Table S1*** *Overview of* *equilibration steps prior to production MD runs with protein backbone (BB) and sidechain (SC) restraints for successive NPT steps. Steps are performed in order from left to right.*
