# Supplementary material for: Alkali metal cations modulate the geometry of different binding sites in HCN4 selectivity filter for permeation or block
Source: J Gen Physiol. 2023 Jul 31;155(10):e202313364. doi: 10.1085/jgp.202313364 (PMC10386491; doi:10.1085/jgp.202313364)
Supplement: Table S2 — shows the summary of simulations with pure cationic solutions. [file JGP_202313364_TableS2.docx]

**Conducted simulations – Table**

|  | **Li^+^** | **K^+^** | | **Rb^+^** | | | **Cs^+^** | | | | |
| --- | --- | --- | --- | --- | --- | --- | --- | --- | --- | --- | --- |
| **U. (mV)** | **-700** | **-500** | **-250** | **-700** | **-500** | **-250** | **-700** | **-500** | **-250** | | **-150** |
| **# Sims** | **4** | **4** | **3** | **1** | **3** | **3** | **2** | **3** | **2** | | **2** |
| $\boldsymbol{\sum}\text{t}_{\text{sim}}$**(μs)** | **2** | **2** | **1.5** | **0.5** | **1.5** | **1.5** | **1** | **1.5** | **1** | | **1.5** |
| **C (mM)** | **900** | | | | | | | | | **500** | |

***Table S2*** *Summary of simulations with pure cationic solutions, with U. as applied membrane potential, # Sims as number of independent simulations,* $\sum\text{t}_{\text{sim}}$ *as total simulation time for a given category and C as cation concentration. All simulations were conducted for the HCN4 pore in the apo-open state.*
