# Supplementary material for: Alkali metal cations modulate the geometry of different binding sites in HCN4 selectivity filter for permeation or block
Source: J Gen Physiol. 2023 Jul 31;155(10):e202313364. doi: 10.1085/jgp.202313364 (PMC10386491; doi:10.1085/jgp.202313364)
Supplement: Table S3 — shows a summary of simulations with mixed-cationic solutions containing Li+. [file JGP_202313364_TableS3.docx]

|  | **Li^+^/K^+^** | | | | **Li^+^/Na^+^** | **Li^+^/Na^+^/K^+^** |
| --- | --- | --- | --- | --- | --- | --- |
| **U. (mV)** | **-700** | **-500** | **-250** | **-150** | **-700** | **-700** |
| **# Sims** | **2** | **3** | **1** | **2** | **1** | **3** |
| $\boldsymbol{\sum}\text{t}_{\text{sim}}$**(μs)** | **1** | **1.5** | **0.5** | **1.5** | **0.5** | **1.5** |
| **C (mM)** | **900** | | | | | |

***Table S3*** *Summary of simulations with mixed-cationic solutions containing Li^+^, with U. as applied membrane potential, # Sims as number of independent simulations,* $\sum\text{t}_{\text{sim}}$ *as total simulation time for a given category and C as cation concentration. All simulations were conducted for the HCN4 pore in the apo-open state.*
