# Supplementary material for: Alkali metal cations modulate the geometry of different binding sites in HCN4 selectivity filter for permeation or block
Source: J Gen Physiol. 2023 Jul 31;155(10):e202313364. doi: 10.1085/jgp.202313364 (PMC10386491; doi:10.1085/jgp.202313364)
Supplement: Table S5 — sshows shows the summary of simulations drawn from Saponaro et al. (2021a) and Bauer (2021). [file JGP_202313364_TableS5.docx]

|  | **Na^+^** | **Na^+^/K^+^** | | **K^+^** | |
| --- | --- | --- | --- | --- | --- |
| **state** | **apo - open** | | | **apo - open** | **holo - closed** |
| **U. (mV)** | **-500** | **-500** | **-700** | **0** | **0** |
| **# Sims** | **2** | **1** | **1** | **1** | **1** |
| $\boldsymbol{\sum}\text{t}_{\text{sim}}$**(μs)** | **2** | **2** | **1.5** | **0.1** | **0.1** |
| **C (mM)** | **900** | | | **150** | |
| **Sim. system** | **Pore region** | | | **Full channel** | |

***Table S5*** *Summary of simulations drawn from* Saponaro et al., 2021a *(pore region) and* Bauer, 2021 *(full channel), with U. as applied membrane potential, # Sims as number of independent simulations,* $\sum\text{t}_{\text{sim}}$ *as total simulation time for a given category and C as cation concentration. All simulations were conducted for the HCN4 pore in the apo-open state. As reference, full channel simulations for the apo-open and the holo-closed state were considered.*
