# Supplementary material for: Alkali metal cations modulate the geometry of different binding sites in HCN4 selectivity filter for permeation or block
Source: J Gen Physiol. 2023 Jul 31;155(10):e202313364. doi: 10.1085/jgp.202313364 (PMC10386491; doi:10.1085/jgp.202313364)
Supplement: Table S6 — shows literature values for the size and hydration number of tested monovalent cations. [file JGP_202313364_TableS6.docx]

**Cationic radii and hydration numbers – Table**

|  | **Li^+^:** | | **Na^+^** | | **K^+^** | | **Rb^+^** | **Cs^+^** |
| --- | --- | --- | --- | --- | --- | --- | --- | --- |
| **effective ionic radius (Å)** | | | | | | | | |
| **CN** | **4** | **6** | **5** | **6** | **6** | **7** | **8** | **8** |
| **Shannon** | **0.59** | **0.76** | **1.0** | **1.02** | **1.38** | **1.46** | **1.61** | **1.74** |
| **Mähler et al.** | **0.6** | **0.79** | **1.02** | **1.07** | **1.38** | **1.46** | **1.64** | **1.74** |
| **Marcus** | **0.74** | | **1.01** | | **1.45** | | **1.50** | **1.79** |
| **hydration number** | | | | | | | | |
| **Mähler et al.** | **4** | | **6** | | **7** | | **8** | **8** |
| **Marcus (min.-max.)** | **4-6** | | **6-8** | | **6-8** | | **5-8** | **7-8** |

***Table S6*** *Literature values for the size and hydration number of tested monovalent cations: Effective ionic radii after Shannon, Mähler et al. (for corresponding coordination numbers CN) and Marcus* *(average values drawn from multiple theoretical and experimental studies; only single MD study is included for Rb^+^), as well as hydration numbers according to Mähler et al. and Marcus (lower and upper limits of hydration numbers).*
